# Development and validation of a sepsis-specific systemic inflammation classification system for mortality prediction

**DOI:** 10.3389/fmed.2026.1852863

**Published:** 2026-06-26

**Authors:** Zhao Wu, Jianliang Cao, Qiang Zhang, Ruyi Lei, Yuepeng Hu, Shuguang Zhang, Chao Lan, Peiyu Wang, Tongwen Sun

**Affiliations:** 1 Department of Emergency, The First Affiliated Hospital of Zhengzhou University, Zhengzhou, Henan, China; 2 General Intensive Care Medicine, The First Affiliated Hospital of Zhengzhou University, Zhengzhou, Henan, China; 3 Department of Thoracic Surgery, The First Affiliated Hospital of Zhengzhou University, Zhengzhou, Henan, China

**Keywords:** intensive care unit, mortality, nutrition, sepsis, systemic inflammation

## Abstract

**Background:**

The role of nutrition and systemic inflammation in the prognosis of sepsis remains unclear. This study aimed to evaluate the predictive value of existing nutrition/inflammation-based indices for mortality in sepsis patients and to develop a sepsis-specific systemic inflammation classification (SSIC) system.

**Methods:**

Using the MIMIC-IV 3.0 database, we investigated and compared the predictive value of 18 nutrition/inflammation-based indices for mortality in sepsis patients admitted to the intensive care unit (ICU). These indices were calculated from biochemical tests and anthropometric measurements at ICU admission. The SSIC system was established by combining existing indices and validated in an independent institutional cohort.

**Results:**

Analysis of 11,577 sepsis patients from the MIMIC-IV 3.0 database revealed that seven of the 18 nutrition/inflammation-based indices had predictive value for in-hospital and in-ICU mortality, albeit with low discriminative ability. The neutrophil-to-lymphocyte ratio (NLR) and glucose-to-lymphocyte ratio (GLR), both of which focus on systemic inflammation, demonstrated the best predictive performance in both indirect and direct comparisons. The SSIC system, which combines NLR and GLR, classified systemic inflammation risk into low, moderate, and high categories. This system improved discriminative ability for mortality and showed consistent predictive value across different subpopulations. Moreover, adding the SSIC system to existing severity scores enhanced their discriminative performance for sepsis mortality. The utility of the SSIC system was validated in an independent institutional cohort of 496 sepsis patients.

**Conclusion:**

This study confirmed the predictive value of 18 nutrition/inflammation-based indices for mortality in sepsis patients. The SSIC system was established and validated as a tool for mortality risk stratification and may be considered for further evaluation alongside established severity scores.

## 1 Introduction

Sepsis, characterized by a dysregulated immune response to infection, is a leading cause of global mortality and frequently requires management in the intensive care unit (ICU) (1). It is associated with high mortality rates, with over 25%−30% of sepsis patients succumbing to the condition, and hospital mortality for septic shock reaching 40%−60% (2). Despite ongoing efforts, the incidence and mortality of sepsis have seen limited reduction over the past decades (3), underscoring the critical need to identify risk factors associated with sepsis outcomes to facilitate early intervention strategies.

Nutrition and systemic inflammation have been shown to influence therapeutic outcomes in chronic diseases and cancers (4–6). Previous studies have suggested that these factors may also affect mortality risk in sepsis patients; however, they employed varying assessment tools and relatively small sample sizes (7–10). Significant gaps remain regarding the optimal tools for assessing nutrition and systemic inflammation and how to integrate them into severity evaluation. As sepsis is marked by a dysregulated systemic inflammatory response and associated with hemodynamic, metabolic, endocrine, and immune abnormalities, substantial nutritional and immune dysregulation occurs (11). Moreover, nutrition and inflammation are closely linked to immunity and interact with each other (12–14). Nutrition and systemic inflammation at the time of treatment could be potential factors for predicting outcomes and guiding clinical decision-making in sepsis patients.

Current assessment of nutrition and systemic inflammation status is primarily based on indices derived from biochemical tests, with or without anthropometric measurements (4, 15). Eighteen nutrition/inflammation-based indices, with broad application and good accessibility (4, 15), are summarized in Supplementary Table 1. Fifteen are biochemical indices: neutrophil-to-lymphocyte ratio (NLR) (7), glucose-to-lymphocyte ratio (GLR) (16), systemic immune-inflammation index (SII) (8), prognostic nutritional index (PNI) (17), albumin-to-globulin ratio (AGR) (18), C-reactive protein-to-albumin ratio (CAR) (19), controlling nutritional status score (CONUT) (20), lymphocyte to C-reactive protein ratio (LCR) (21), lymphocyte C-reactive protein score (LCS) (22), lymphocyte-to-monocyte ratio (LMR) (23), modified Glasgow prognostic score (mGPS) (24), neutrophil-to-platelet ratio (NPR) (9), platelet-to-lymphocyte ratio (PLR) (25), systemic inflammation response index (SIRI) (10), and systemic nutrition-inflammation index (SNII) (15). The biochemical-anthropometric indices included the advanced lung cancer inflammation index (ALI) (4), geriatric nutritional risk index (GNRI) (26), and the modified geriatric nutritional risk index (mGNRI) (27). Among these, PNI (17) and NLR (28) have been associated with sepsis development, while NLR (7), GLR (16), and SII (8) have been shown to predict sepsis mortality. However, the predictive values of these nutrition/inflammation-based indices for sepsis mortality have not been systematically assessed and compared in large cohorts. Furthermore, these indices are mostly continuous variables, highlighting the need for an effective classification system to facilitate clinical application.

In summary, this study primarily aimed to investigate and compare the predictive value of 18 nutrition/inflammation-based indices at ICU admission for mortality in sepsis patients. Furthermore, we combined indices with good predictive value to develop a sepsis-specific classification system that assesses nutrition and/or systemic inflammation status, thereby improving mortality prediction.

## 2 Methods and materials

### 2.1 Study design

**Figure 1:**
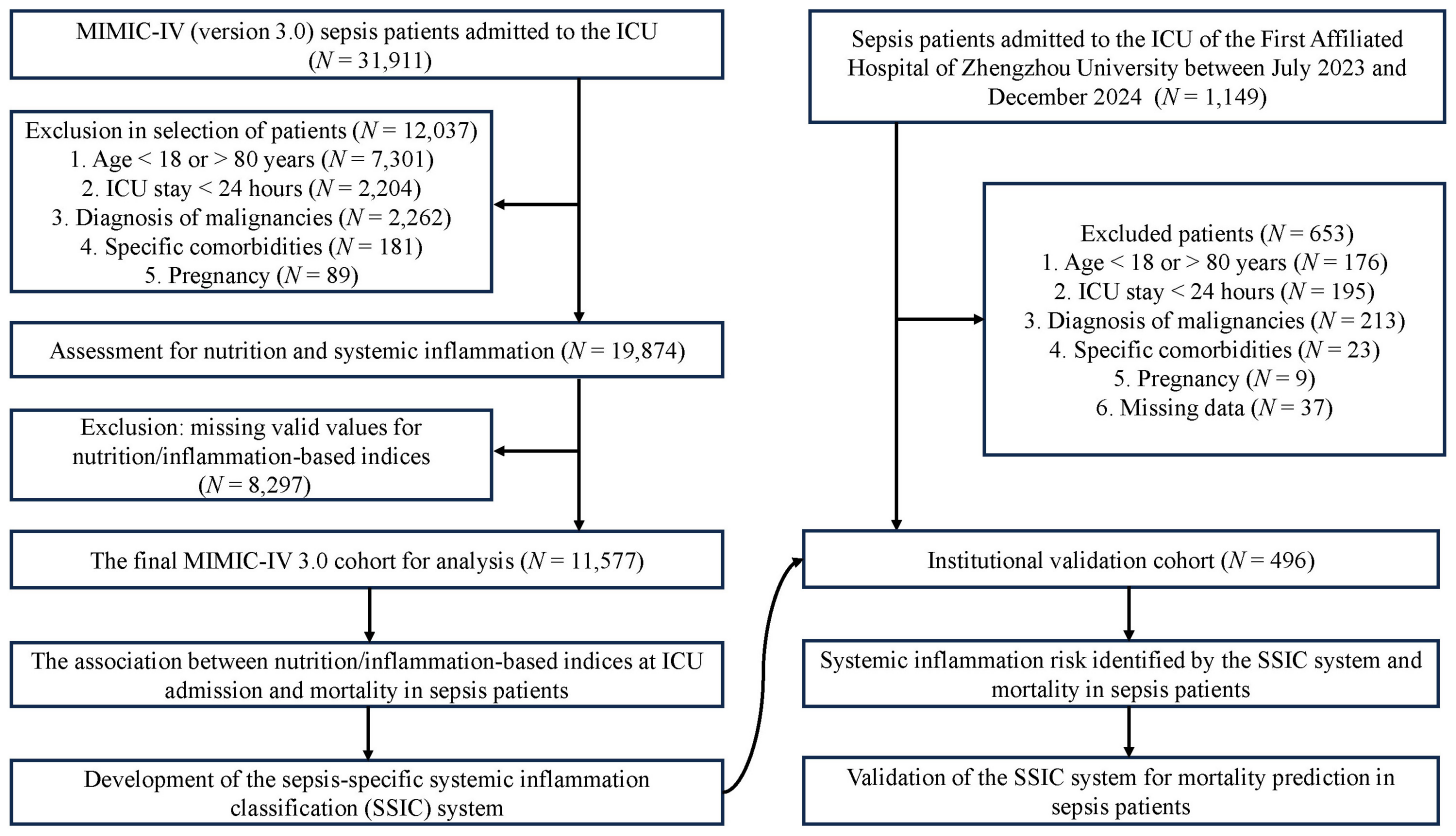
Study design and patient recruitment flowchart for the MIMIC-IV 3.0 cohort and the institutional validation cohort. ICU, intensive care unit; MIMIC, Medical Information Mart for Intensive Care database; SSIC, sepsis-specific systemic inflammation classification.

This study involved analyses of the Medical Information Mart for Intensive Care database (MIMIC-IV version 3.0, referred to as the MIMIC-IV 3.0 cohort) and an independent institutional validation cohort, as illustrated in Figure 1. We analyzed the MIMIC-IV 3.0 cohort to investigate and compare the predictive values of 18 nutrition/inflammation-based indices for mortality in sepsis patients admitted to the ICU. A sepsis-specific classification system was developed by combining indices with optimal predictive performance. Access to the MIMIC-IV database was approved by PhysioNet/MIT on January 31, 2024, and authorized to author Wu Zhao after completion of CITI training (Record ID: 60828152) and signing of the data use agreement. The data use period began on January 31, 2024, and is ongoing; all raw data will be permanently deleted within 30 days after study completion. The institutional cohort, comprising sepsis patients treated in the ICU of the First Affiliated Hospital of Zhengzhou University between July 2023 and December 2024, was analyzed to validate the performance of sepsis-specific classification system in predicting sepsis mortality. Data collection for the institutional cohort was prospective, while analysis was conducted retrospectively. The study protocol was approved by the Ethics Committee Board of the First Affiliated Hospital of Zhengzhou University (Approval No. 2024-KY-1909-001). Informed consent was obtained from all patients for the use of their data in institutional database. The study adhered to the Strengthening the Reporting of Observational Studies in Epidemiology (STROBE) Statement guidelines (29).

### 2.2 MIMIC-IV 3.0 and data extraction

The MIMIC-IV 3.0 database is a freely accessible resource containing data from over 90,000 ICU admissions at the Beth Israel Deaconess Medical Center in Boston, Massachusetts, from 2008 to 2022 (30). It includes comprehensive data such as demographics, comorbidities, vital signs, severity scores, laboratory results, and diagnoses coded using the International Classification of Diseases, Ninth and Tenth Revision (ICD-9 and ICD-10). Data extraction was performed using PostgresSQL and Navicat Premium software via Structured Query Language (SQL). Baseline data were extracted based on measurements or assessments taken immediately after ICU admission. The extracted severity score included sequential organ failure assessment (SOFA) score, acute physiology score III (APS III), inflammatory response syndrome (SIRS) score, simplified acute physiology score II (SAPS II), and Oxford acute severity of illness score (OASIS), and Glasgow coma scale (GCS). Follow-up began on the date of ICU admission and ended at hospital discharge or death.

### 2.3 Study population

Patients were diagnosed with sepsis according to the Sepsis 3.0 diagnostic criteria (31, 32). The detailed approach and SQL code for identifying sepsis patients from the MIMIC-IV database are provided in Supplementary Data 1. Key criteria included: (1) clinical suspicion of infection, determined by the earlier timestamp of antibiotic administration and cultures within a specified timeframe; (2) occurrence of end-organ damage, identified by an increase in SOFA score of 2 points or more; (3) sepsis onset time defined as the earlier of time-suspicion and time-SOFA, provided time-SOFA occurred no more than 48 h before or 24 h after time-suspicion; otherwise, the patient was not classified as having sepsis. Exclusion criteria were: (1) age <18 or > 80 years; (2) ICU stay <24 h; (3) diagnosis of malignancies; (4) rheumatic and immune system diseases or other specific comorbidities; (5) pregnancy; and (6) missing critical parameters.

### 2.4 Nutrition/inflammation-based indices

For both MIMIC-IV 3.0 and institutional cohort cohorts, nutrition/inflammation-based indices were evaluated from routine biochemical tests and anthropometric measurements conducted immediately after ICU admission (Supplementary Table 1). This time point was selected based on our aim to assess the predictive value of these indices at ICU admission for sepsis mortality, as well as their utility in integrating with current severity assessments at the same time point. Biochemical parameters included serum globulin, serum albumin, C-reactive protein, total cholesterol, serum glucose, hemoglobin, total neutrophils, total lymphocytes, total monocytes, and total platelets in peripheral blood. Anthropometric measurements included present weight, ideal weight, and body mass index. Ideal weight was calculated from height and sex with the Lorentz equations (33). This study investigated 18 nutrition/inflammation-based indices that have been previously established and validated in human diseases. Among them, four indices focused on nutrition assessment (AGR (18), PNI (17), CONUT (20), and GNRI (26)), nine indices focused on systemic inflammation assessment (NLR (7), GLR (16), SII (8), LCR (21), LCS (22), LMR (23), NPR (9), PLR (25), and SIRI (10)),and the remaining five indices comprehensively assessed both nutrition and systemic inflammation (CAR (19), mGPS (24), SNII (15), ALI (4), and mGNRI (27)). At the participant recruitment stage, these 18 indices were calculated for all patients according to the formulas shown in Supplementary Table 1. Missing basic biochemical parameters or anthropometric measurements led to missing data for the calculation of these indices. Participants were included if they had at least one valid value for any of the 18 indices; otherwise, they were excluded.

### 2.5 Development of the sepsis-specific classification system for nutrition and (or) systemic inflammation

We initially evaluated the predictive power of existing nutrition/inflammation-based indicators for sepsis mortality in the MIMIC-IV 3.0 cohort. To enhance predictive performance, we combined indices with optimal predictive value to establish a sepsis-specific classification system. The system was established by scoring component elements based on optimal cut-offs derived from receiver operating characteristic (ROC) curves using the Youden index. This system classified nutrition and (or) systemic inflammation risk into low, moderate, and high categories. Its predictive efficacy for sepsis mortality was assessed in both the MIMIC-IV 3.0 database and the institutional validation cohort.

### 2.6 Endpoints

The primary outcome was in-hospital mortality, and the secondary endpoint was in-ICU mortality. Given the use of Cox proportional hazards regression models for survival analysis, mortality at specific time points was not separately investigated as an endpoint.

### 2.7 Sample size assessment

The sample size for the institutional validation cohort was determined based on the analysis outcomes of the MIMIC-IV 3.0 cohort, following published methods (34). In-hospital mortality was selected as the endpoint. The percentage of participants assessed as high vs. low-moderate systemic nutrition-inflammation risk were used for calculation. The significance level (α) was set at 0.05, with a statistical power of 0.95.

### 2.8 Statistical analysis

Categorical data are presented as frequencies (percentages), and continuous data as means (standard deviations, SDs) or medians (interquartile ranges, IQRs). Because the laboratory values are directly derived from clinical records in both cohorts, possible extreme values and outliers are clinically plausible and were retained in the analysis to avoid selection bias. Group differences were evaluated using ANOVA, Pearson's chi-squared tests, or Kruskal–Wallis tests. ROC curves were used to assess the discriminative ability of variables for sepsis mortality, with the area under the curve (AUC) indicating performance. Calibration curves were created to illustrate the calibration of established models for sepsis mortality. Multivariable analysis using logistic regression models or Cox proportional hazards regression models was conducted to demonstrate independent associations between systemic nutrition-inflammation indicators and sepsis mortality. Odds ratios (ORs) and hazard ratios (HRs) with 95% confidence intervals (CIs) are reported. Significant collinearity among variables included in multivariable analysis was identified by Tolerance <0.1 or Variance Inflation Factor >10. Due to substantial missing data for several indices in the MIMIC-IV 3.0 cohort, these 18 nutrition/inflammation-based indices were first individually incorporated into ROC curve and multivariable analyses. Markov Chain Monte Carlo (MCMC) multiple imputation methods were then used to impute the missing values of indices with a missing proportion of less than 70% (35, 36). These indices were directly compared using ROC curve and multivariable analysis in complete case analysis and then compared in five multiple imputation models as a sensitive analysis. A binary logistic regression model was constructed to combine the established sepsis-specific classification system and the existing severity scores for predicting sepsis mortality. Both predictors were included as covariates to estimate their joint association with the outcome, generating an individualized predicted probability. Subgroup analysis of clinical characteristics was performed to identify vulnerable populations at risk from nutrition and systemic inflammation effects. A two-sided *P*-value of less than 0.05 was considered statistically significant. All analyses were performed using IBM SPSS Statistics for Windows (version 22.0, IBM Corp., Armonk, NY, USA) and the R programming environment (version 4.2.0, R Core Team, Vienna, Austria).

## 3 Results

### 3.1 MIMIC-IV 3.0 cohort

#### 3.1.1 Patient characteristics

A review of the MIMIC-IV 3.0 database identified 31,911 sepsis patients admitted to the ICU. Initial assessment excluded 12,037 patients for reasons detailed in Figure 1. An additional 8,297 patients were excluded due to missing data required for calculation of at least one of the 18 nutrition/inflammation-based indices. The remaining 11,577 patients formed the MIMIC-IV 3.0 cohort (Table 1). The mean age was 60.4 years (SD: 13.8), with 39.1% were female. Mechanical ventilation was used in 10,427 (90.1%) patients, and continuous renal replacement therapy was administrated to 1,421 (12.3%) patients. In-hospital mortality occurred in 2,281 (19.7%) patients, and in-ICU mortality in 1,633 (14.1%) patients. Data integrity for demographics, comorbidities, vital signs, severity scores, and interventions was 100%. Patients excluded due to missing systemic nutrition-inflammation data had fewer comorbidities, better vital signs, better severity scores, received fewer medical interventions, and had lower mortality rates than those included in the MIMIC-IV 3.0 cohort (Supplementary Table 2).

**Table 1:** Characteristics of sepsis patients in the MIMIC-IV 3.0 cohort.

Characteristics	Total cohort (*N* = 11,577)	Missing data (%)
*Demographic data*		
Age, years	60.4 ± 13.8	0
Gender (female)	4,523 (39.1)	0
Body mass index, kg/m^2^	28.7 (24.7–33.9)	0
*Comorbidities*		
Cardiovascular	7,186 (62.1)	0
Liver	2,082 (18.0)	0
Renal	2,214 (19.1)	0
Diabetes	3,782 (32.7)	0
COPD	1,166 (10.1)	0
Stroke	835 (7.2)	0
*Vital signs*		
MAP, mmHg	79 (69–93)	0
Heart rate,/min	89 (78–105)	0
Respiratory rate,/min	19 (16–24)	0
SpO_2_, %	98 (95–100)	0
Temperature, °C	36.8 (36.5–37.2)	0
*Severity scores*		
SOFA score	6 (4–9)	0
APS III score	49 (36–66)	0
SIRS score	3 (2–3)	0
SAPS II score	38 (30–48)	0
OASIS score	34 (28–40)	0
GCS score	15 (14–15)	0
*Interventions*		
Mechanical ventilation	10,427 (90.1)	0
Ventilation duration, hours	58.6 (21.3–143)	0
CRRT	1,421 (12.3)	0
*Nutrition/inflammation–based indicators*		
*Nutrition-inflammation-based items*		
Serum albumin, g/L	3.0 (2.5–3.4)	16.1
Serum globulin, g/L	2.5 (2.0–3.1)	90.5
C–reactive protein, mg/L	100 (32.5–180)	94.0
Total cholesterol, mg/dL	125 (93–165)	87.3
Serum glucose, mg/dL	131 (107–173)	0.01
Hemoglobin, g/dL	10.3 (8.7–12.1)	0.34
Total neutrophils, × 10^3^/μl	9.46 (6.08–14.1)	38.4
Total lymphocytes, × 10^3^/μl	1.14 (0.67–1.77)	38.4
Total monocytes, × 10^3^/μl	0.63 (0.34–1.02)	58.8
Total platelet, × 10^3^/μl	174 (116–246)	0.52
*Nutrition-inflammation-based indices*		
AGR	1.12 (0.76–1.55)	90.5
NLR	7.94 (4.39–15.2)	38.8
PLR, × 10	15.1 (8.18–27.5)	38.9
NPR, × 10^−2^	5.59 (3.44–8.79)	38.8
LMR	1.72 (0.93–3.46)	59.7
SIRI	5.40 (2.08–13.4)	59.8
SII, × 10^2^	13.3 (6.26–28.9)	39.1
SNII	168 (87–310)	95.4
PNI	32.2 (20.8–38.9)	40.1
GNRI	99.3 (88.5–112)	30.0
ALI	9.42 (4.53–17.7)	67.8
GLR, × 10^2^	1.24 (0.71–2.30)	38.6
CAR, × 10	3.71 (1.11–6.43)	94.8
LCR	93.3 (45.9–330)	96.0
mGNRI	57.1 (48.3–67.7)	96.1
mGPS	2 (1–2)	94.8
LCS	2 (1–2)	95.9
COUNT	6.0 (3.0–8.0)	91.8
*Outcomes*		
In–hospital mortality	2,281 (19.7)	0
In-ICU mortality	1,633 (14.1)	0
Length of ICU stay, day	4.81 (2.34–10.0)	0
Length of hospital stay, day	12.9 (7.18–22.8)	0

Data are mean ± standard deviation, number (percentage), or median (interquartile range). AGR, albumin-to-globulin ratio; ALI, advanced lung cancer inflammation index; APS III, acute physiology score III; CAR, C-reactive protein-to-albumin ratio; COPD, chronic obstructive pulmonary disease; CONUT, controlling nutritional status score; CRRT, continuous renal replacement therapy; GCS, Glasgow coma scale; GLR, glucose-to-lymphocyte ratio; GNRI, geriatric nutritional risk index; ICU, intensive care unit; LCR, lymphocyte to C-reactive protein ratio; LCS, lymphocyte C-reactive protein score; LMR, lymphocyte-to-monocyte ratio; MAP, mean arterial pressure; mGNRI, modified geriatric nutritional risk index; mGPS, modified Glasgow prognostic score; NLR, neutrophil-to-lymphocyte ratio; NPR, neutrophil-to-platelet ratio; OASIS, Oxford acute severity of illness score; PLR, platelet-to-lymphocyte ratio; PNI, prognostic nutritional index; SAPS II, simplified acute physiology score II; SII, systemic immune-inflammation index; SIRI, systemic inflammation response index; SIRS, systemic inflammatory response syndrome; SNII, systemic nutrition-inflammation index; SOFA, sequential organ failure assessment; SpO_2_, peripheral capillary oxygen saturation.

#### 3.1.2 Nutrition/inflammation-based indices

The nutrition/inflammation-based items and indices for the MIMIC-IV 3.0 cohort are detailed in Table 1. As patients were included if they had at least one valid value among the 18 indices, missing data varied widely across basic biochemical indicators, from 0.01% for serum glucose to 94.0% for C-reactive protein. Consequently, indices involving C-reactive protein (CAR, LCR, mGNRI, mGPS, and LCS) had data missing rates exceeding 90%. Similarly high missing rates were observed for indices involving serum globulin and total cholesterol (AGR, COUNT). For ten indices with data missing rates less than 70% (NLR, PLR, NPR, LMR, SIRI, SII, PNI, GNRI, ALI, and GLR), five multiple imputation models were established to impute the missing values, as reported in Supplementary Table 3.

#### 3.1.3 Nutrition/inflammation-based indices and mortality: indirect comparisons

Based on ROC curve analysis, most nutrition/inflammation-based indices showed predictive value for in-hospital and in-ICU mortality in sepsis patients (Table 2), albeit with varying associations. Discriminative ability, reflected by AUC values, was generally low, with the best performance observed for the NLR and GLR. Among nutrition/inflammation-based items, serum albumin, serum glucose, total neutrophils, total lymphocytes, total monocytes, and total platelets predicted sepsis mortality, with the highest performance for total lymphocytes.

**Table 2:** Discriminative ability of nutrition/inflammation-based indicators for predicting sepsis mortality in the MIMIC-IV 3.0 cohort.

Parameters	In-hospital mortality		In-ICU mortality		Association
	AUC (95% CI)	*P* value	AUC (95% CI)	*P* value	
*Nutrition/inflammation-based indices*					
AGR	0.505 (0.464–0.545)	0.82	0.529 (0.475–0.583)	0.27	Positive
NLR	0.643 (0.627–0.660)	<0.001	0.643 (0.624–0.663)	<0.001	Positive
PLR	0.561 (0.543–0.578)	<0.001	0.560 (0.539–0.581)	<0.001	Positive
NPR	0.599 (0.579–0.619)	<0.001	0.593 (0.576–0.611)	<0.001	Positive
LMR	0.612 (0.591–0.632)	<0.001	0.608 (0.584–0.632)	<0.001	Negative
SIRI	0.615 (0.594–0.636)	<0.001	0.604 (0.578–0.629)	<0.001	Positive
SII	0.585 (0.568–0.603)	<0.001	0.592 (0.572–0.612)	<0.001	Positive
SNII	0.589 (0.525–0.653)	0.004	0.567 (0.490–0.645)	0.070	Negative
PNI	0.522 (0.506–0.539)	0.010	0.513 (0.494–0.532)	0.20	Positive
GNRI	0.530 (0.514–0.545)	<0.001	0.513 (0.496–0.530)	0.13	Negative
ALI	0.614 (0.593–0.635)	<0.001	0.604 (0.581–0.627)	<0.001	Negative
GLR	0.625 (0.608–0.641)	<0.001	0.623 (0.604–0.643)	<0.001	Positive
CAR	0.554 (0.501–0.607)	0.049	0.560 (0.498–0.622)	0.059	Positive
LCR	0.594 (0.534–0.653)	0.002	0.593 (0.523–0.662)	0.002	Negative
mGNRI	0.563 (0.507–0.619)	0.036	0.580 (0.523–0.638)	0.013	Positive
mGPS	0.563 (0.511–0.615)	0.022	0.555 (0.495–0.615)	0.082	Positive
LCS	0.617 (0.561–0.674)	<0.001	0.605 (0.542–0.668)	0.002	Positive
COUNT	0.560 (0.514–0.607)	0.010	0.535 (0.475–0.595)	0.23	Positive
*Nutrition/inflammation-based items*					
Serum albumin	0.564 (0.549–0.578)	<0.001	0.556 (0.539–0.572)	<0.001	Negative
Serum globulin	0.581 (0.522–0.640)	0.007	0.550 (0.477–0.623)	0.19	Negative
C–reactive protein	0.539 (0.490–0.588)	0.13	0.549 (0.493–0.606)	0.093	Positive
Total cholesterol	0.540 (0.501–0.580)	0.037	0.516 (0.468–0.564)	0.49	Negative
Serum glucose	0.542 (0.528–0.556)	<0.001	0.560 (0.544–0.576)	<0.001	Positive
Hemoglobin	0.537 (0.523–0.551)	<0.001	0.502 (0.487–0.518)	0.75	Negative
Total neutrophils	0.575 (0.558–0.593)	<0.001	0.593 (0.572–0.613)	<0.001	Positive
Total lymphocytes	0.618 (0.602–0.635)	<0.001	0.607 (0.587–0.627)	<0.001	Negative
Total monocytes	0.542 (0.521–0.564)	<0.001	0.534 (0.509–0.559)	0.005	Positive
Total platelet	0.537 (0.523–0.551)	<0.001	0.522 (0.506–0.538)	0.004	Negative

Receiver Operating Characteristic (ROC) curves were used to individually assess discriminative ability of nutrition/inflammation-based indicators for predicting mortality in sepsis patients. Area under the curve (AUC) and its 95% confidence interval (CI) were reported. AGR, albumin-to-globulin ratio; ALI, advanced lung cancer inflammation index; CAR, C-reactive protein-to-albumin ratio; CONUT, controlling nutritional status score; CRRT, continuous renal replacement therapy; GLR, glucose-to-lymphocyte ratio; GNRI, geriatric nutritional risk index; ICU, intensive care unit; LCR, lymphocyte to C-reactive protein ratio; LCS, lymphocyte C-reactive protein score; LMR, lymphocyte-to-monocyte ratio; mGNRI, modified geriatric nutritional risk index; mGPS, modified Glasgow prognostic score; NLR, neutrophil-to-lymphocyte ratio; NPR, neutrophil-to-platelet ratio; PLR, platelet-to-lymphocyte ratio; PNI, prognostic nutritional index; SII, systemic immune-inflammation index; SIRI, systemic inflammation response index; SNII, systemic nutrition-inflammation index.

Detailed results of univariable and multivariable analyses for predictive factors of in-hospital and in-ICU mortality in sepsis patients are shown in Supplementary Tables 4, 5, respectively. Baseline clinical characteristics independently associated with either in-hospital or in-ICU mortality included age, comorbidities (cardiovascular disease, liver disease, renal disease, and COPD), vital signs (respiratory rate, SpO_2_, and temperature), and severity scores (SOFA, APS III, SIRS, OASIS, and GCS). No significant collinearity was detected among these variables (Supplementary Table 6); accordingly, they were selected as adjustment factors to assess the independent association between nutrition/inflammation-based indicators and sepsis mortality.

After adjustment, NLR, PLR, NPR, SIRI, SII, GLR, and LCS were demonstrated to be independently associated with in-hospital mortality (Supplementary Table 4). Similarly, NLR, PLR, SII, GLR, and LCS were independently associated with in-ICU mortality (Supplementary Table 5). In contrast, indices focused solely on nutrition assessment or combined assessment of nutrition and systemic inflammation showed no independent predictive value. Among nutrition/inflammation-based items, serum glucose and total neutrophils were independently associated with in-hospital and in-ICU mortality, while total lymphocytes showed no significant association.

#### 3.1.4 Nutrition/inflammation-based and mortality: direct comparisons

Eight nutrition/inflammation-based indices involving C-reactive protein, serum globulin, or total cholesterol were excluded from direct comparisons due to high missing rates. Final direct comparisons involved the remaining 10 indices (NLR, PLR, NPR, LMR, SIRI, SII, PNI, GNRI, ALI, and GLR) in a sample of 2,360 patients in complete case analysis and in samples of 11,577 in multiple imputation models. ROC curve analysis showed that NLR and GLR had the highest AUC values for predicting in-hospital and in-ICU mortality (Table 3). Multivariable analysis using logistic regression models and Cox proportional hazards regression models confirmed that NLR and GLR retained optimal independent predictive value for both endpoints (Table 3). In sensitive analysis with five multiple imputation models, NLR and GLR still showed the highest AUC values for predicting in-hospital and in-ICU mortality (Supplementary Table 7), and they were the only two indices independently associated with sepsis mortalities (Supplementary Table 8).

**Table 3:** Predictive value of 9 nutrition/inflammation-based indices for sepsis mortality in the MIMIC-IV 3.0 cohort (*N* = 2360).

Indices	Comparisons	Multivariable analysis^1^				Receiver operating characteristic curve	
		Logistic regression models		Cox proportional hazards regression models			
		OR (95% CI)	*P* value	HR (95% CI)	*P* value	AUC (95% CI)	*P* value
*In-hospital mortality*							
NLR	Per 1 unit	1.014 (1.004–1.024)	0.006	1.011 (1.004–1.017)	0.001	0.584 (0.558–0.611)	<0.001
PLR	Per 10 unit	0.999 (0.994–1.003)	0.63	0.999 (0.997–1.002)	0.59	0.519 (0.491–0.546)	0.16
NPR	Per 0.01 unit	1.011 (1.001–1.022)	0.024	1.004 (0.999–1.010)	0.10	0.566 (0.539–0.593)	<0.001
LMR	Per 1 unit	1.008 (0.989–1.027)	0.44	1.009 (0.995–1.024)	0.19	0.568 (0.541–0.595)	<0.001
SIRI	Per 1 unit	1.004 (0.999–1.009)	0.12	1.003 (1.000–1.006)	0.036	0.563 (0.536–0.590)	<0.001
SII	Per 100 unit	0.997 (0.993–1.001)	0.16	0.998 (0.995–1.001)	0.15	0.525 (0.498–0.552)	0.063
PNI	Per 1 unit	1.000 (0.997–1.003)	0.95	1.000 (0.997–1.002)	0.74	0.576 (0.549–0.603)	<0.001
GNRI	Per 1 unit	0.997 (0.992–1.002)	0.24	0.999 (0.996–1.003)	0.78	0.529 (0.503–0.556)	0.028
ALI	Per 1 unit	1.000 (1.000–1.000)	0.50	1.000 (1.000–1.000)	0.69	0.577 (0.550–0.604)	<0.001
GLR	Per 100 unit	1.048 (1.013–1.084)	0.006	1.023 (1.010–1.037)	0.001	0.590 (0.564–0.616)	<0.001
*In–ICU mortality*							
NLR	Per 1 unit	1.011 (1.001–1.021)	0.032	1.009 (1.001–1.017)	0.019	0.572 (0.542–0.601)	<0.001
PLR	Per 10 unit	0.998 (0.993–1.002)	0.36	0.998 (0.995–1.001)	0.13	0.516 (0.486–0.545)	0.29
NPR	Per 0.01 unit	1.004 (0.996–1.013)	0.30	1.005 (0.999–1.012)	0.081	0.565 (0.535–0.594)	<0.001
LMR	Per 1 unit	1.013 (0.993–1.033)	0.21	1.013 (0.999–1.027)	0.077	0.549 (0.519–0.578)	0.001
SIRI	Per 1 unit	1.003 (0.998–1.007)	0.25	1.004 (1.001–1.008)	0.016	0.552 (0.521–0.582)	<0.001
SII	Per 100 unit	0.998 (0.994–1.003)	0.45	0.998 (0.995–1.001)	0.23	0.526 (0.497–0.556)	0.073
PNI	Per 1 unit	1.000 (0.996–1.003)	0.83	0.999 (0.997–1.002)	0.49	0.557 (0.527–0.586)	<0.001
GNRI	Per 1 unit	1.003 (0.998–1.008)	0.30	1.000 (0.996–1.004)	0.91	0.500 (0.471–0.529)	1.00
ALI	Per 1 unit	1.000 (1.000–1.000)	0.55	1.000 (1.000–1.000)	0.69	0.569 (0.540–0.598)	<0.001
GLR	Per 100 unit	1.049 (1.015–1.084)	0.004	1.023 (1.005–1.041)	0.014	0.582 (0.553–0.611)	<0.001

^1^Ten nutrition/inflammation-based indices were simultaneously entered into multivariate analyses. Results are reported as odds ratios (ORs) or hazard ratios (HRs) with 95% confidence intervals (CIs). ALI, advanced lung cancer inflammation index; AUC, area under the curve; GLR, glucose-to-lymphocyte ratio; GNRI, geriatric nutritional risk index; LMR, lymphocyte-to-monocyte ratio; NLR, neutrophil-to-lymphocyte ratio; NPR, neutrophil-to-platelet ratio; PLR, platelet-to-lymphocyte ratio; PNI, prognostic nutritional index; SII, systemic immune-inflammation index; SIRI, systemic inflammation response index.

#### 3.1.5 Development of the sepsis-specific systemic inflammation classification (SSIC) system

To enhance predictive performance, we combined existing nutrition/inflammation-based indicators to establish a sepsis-specific classification system. C-reactive protein, serum globulin, and total cholesterol were excluded due to high missing rates. Based on previous comparisons, NLR and GLR—both focused on the assessment of systemic inflammation—showed optimal predictive value for in-hospital and in-ICU mortality, outperforming indices that assessed nutrition status or combined nutrition and inflammation status. Their constituent items (serum glucose, total neutrophils, and total lymphocytes) also demonstrated strong predictive performance. Thus, we proposed two candidates: one combining NLR and GLR, and another combining the three basic items (Table 4). After excluding patients without required data, 7,088 participants were used for developing the sepsis-specific classification system.

**Table 4:** Systemic inflammation classification systems and sepsis mortality in the MIMIC-IV 3.0 cohort (*N* = 7088).

Classification systems	Risk comparisons	In-hospital mortality						In-ICU mortality					
		Logistic regression models^5^		Cox proportional hazardsregression models^5^		Receiver operating characteristic curve		Logistic regression models^5^		Cox proportional hazards regression models^5^		Receiver operating characteristic curve	
		OR (95% CI)	*P* value	HR (95% CI)	*P* value	AUC (95% CI)	*P* value	OR (95% CI)	*P* value	HR (95% CI)	*P* value	AUC (95% CI)	*P* value
Classification of NLR ^1^	High vs. low	1.73 (1.51–1.98)	<0.001	1.53 (1.37–1.71)	<0.001	0.608 (0.592–0.624)	<0.001	1.73 (1.47–2.03)	<0.001	1.38 (1.21–1.59)	<0.001	0.603 (0.583–0.622)	<0.001
Classification of GLR^2^	High vs. low	1.54 (1.35–1.76)	<0.001	1.39 (1.25–1.54)	<0.001	0.599 (0.582–0.616)	<0.001	1.65 (1.41–1.93)	<0.001	1.42 (1.25–1.62)	<0.001	0.598 (0.578–0.618)	<0.001
Combination of NLR and GLR (SSIC)^3^	Low (reference)	–	–	–	–	0.641 (0.625–0.657)	<0.001	–	–	–	–	0.642 (0.623–0.660)	<0.001
	Moderate	1.40 (1.17–1.66)	<0.001	1.26 (1.10–1.46)	0.001			1.57 (1.28–1.92)	<0.001	1.33 (1.12–1.58)	0.001		
	High	2.02 (1.72–2.38)	<0.001	1.73 (1.51–1.97)	<0.001			2.14 (1.76–2.59)	<0.001	1.66 (1.40–1.96)	<0.001		
Components of NLR and GLR^4^	Low (reference)	–	–	–	–	0.627 (0.611–0.642)	<0.001	–	–	–	–	0.642 (0.624–0.660)	<0.001
	Moderate	1.43 (1.16–1.76)	0.001	1.30 (1.09–1.56)	0.004			1.73 (1.33–2.25)	<0.001	1.38 (1.08–1.74)	0.010		
	High	1.80 (1.46–2.22)	<0.001	1.72 (1.44–2.05)	<0.001			2.41 (1.85–3.13)	<0.001	1.84 (1.45–2.32)	<0.001		

^1^The NLR was categorized based on the optimal cut-offs for predicting in-hospital mortality and defined systemic inflammation risk as high (NLR >8.89) and low (NLR ≤ 8.89). ^2^The GLR was categorized based on the optimal cut-offs for predicting in-hospital mortality and defined systemic inflammation risk as high (GLR >167) and low (GLR ≤ 167). ^3^The optimal cut-offs of NLR and GLR for predicting in-hospital mortality were initially used to classify systemic inflammation risk as high or low, assigning a score of 1 and 0, respectively. Subsequently, the scores of NLR and GLR were summed to obtain a total score ranging from 0 to 2. The final systemic inflammation risk was defined as low (score 0), moderate (score 1), or high (score 2) based on the combined scores of NLR and GLR. ^4^The basic components of NLR and GLR include total neutrophils, total lymphocytes, and serum glucose. The optimal cut-offs of these components for predicting in-hospital mortality were 11.66 × 10^3^/μl, 1.02 × 10^3^/μl, and 148 mg/dL, respectively. These cut-offs were initially used to classify systemic inflammation risk as high or low, assigning a score of 1 and 0, respectively. The scores of the three components were then summed to obtain a total score ranging from 0 to 3. The final systemic inflammation risk was defined as low (score 0), moderate (score 1), or high (score 2–3) based on the combined score of three components. ^5^Multivariable analyses were conducted using logistic regression models or Cox proportional hazards regression models with the backward conditional methods. These models included adjustments for demographics, comorbidities, vital signs, and severity scores which were demonstrated as independent risk factors for mortality as shown in Supplementary Tables 4 and 5. AUC, area under the curve; CI, confidence interval; GLR, glucose-to-lymphocyte ratio; HR, hazard ratio; ICU, intensive care unit; NLR, neutrophil-to-lymphocyte ratio; OR, odds ratio; SSIC, sepsis-specific systemic inflammation classification.

The combination of NLR and GLR demonstrated superior predictive performance for in-hospital mortality and comparable performance for in-ICU mortality compared to the combination of the three basic items (Table 4). Both composite systems improved predictive performance over individual NLR or GLR classifications. Furthermore, after adjustment for clinical predictors of mortality presented in Supplementary Tables 4, 5, both candidate systems remained independently associated with sepsis mortality. Therefore, the combination of NLR and GLR was used to develop the sepsis-specific classification system. As NLR and GLR focus on the assessment of systemic inflammation, this system was finally named as the sepsis-specific systemic inflammation classification (SSIC) system. The optimal cut-offs for NLR and GLR for predicting in-hospital mortality were used to classify systemic inflammation risk as high (NLR > 8.89, GLR > 167) or low (NLR ≤ 8.89, GLR ≤ 167), with scores of 1 and 0, respectively. Scores for NLR and GLR were then summed, yielding a total score from 0 to 2. Final systemic inflammation risk was defined as low (score 0), moderate (score 1), or high (score 2).

#### 3.1.6 Subgroup analysis of the SSIC system

**Figure 2:**
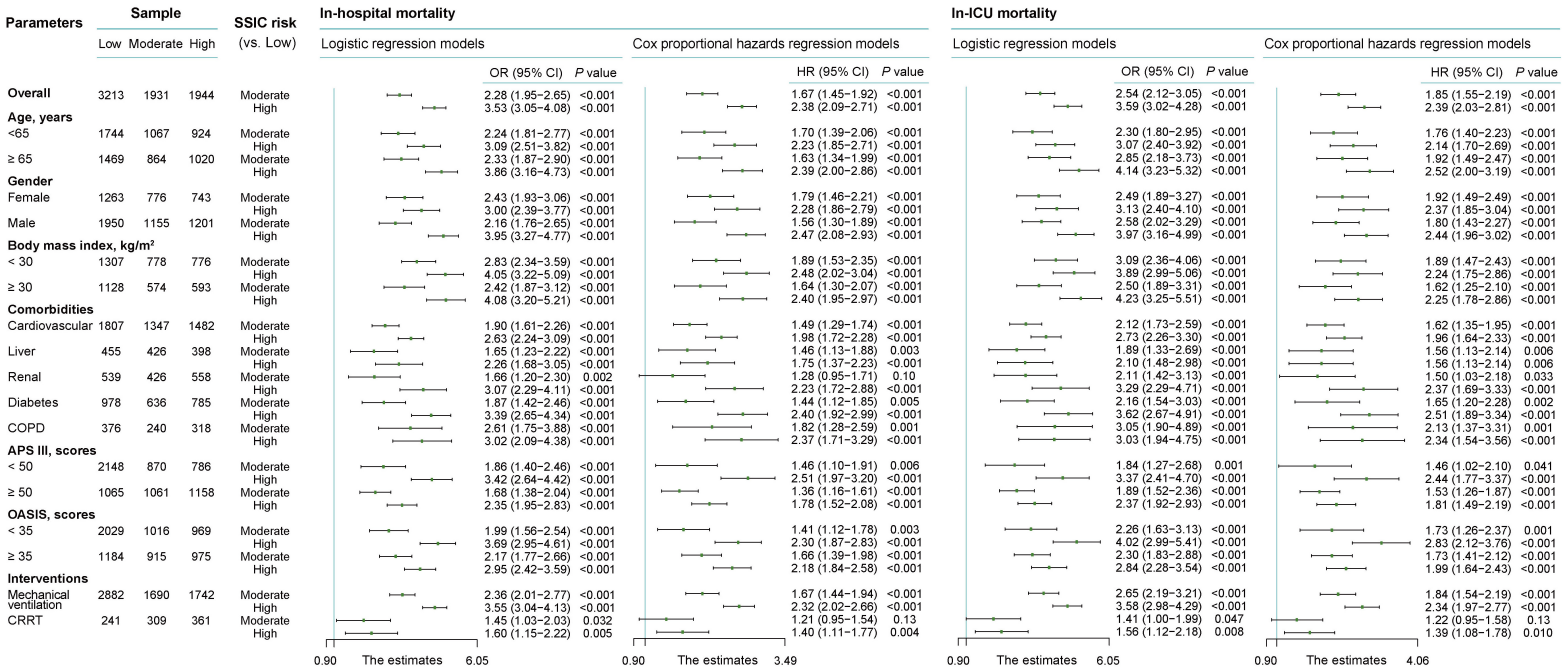
Forest plots of subgroup analysis for association between systemic inflammation risk classified by the SSIC system and sepsis mortality in the MIMIC-IV 3.0 cohort. APS III, acute physiology score III; C, confidence interval; COPD, chronic obstructive pulmonary disease; CRRT, continuous renal replacement therapy; HR, hazard ratio; ICU, intensive care unit; OASIS, Oxford acute severity of illness score; OR, odds ratio; SSIC, sepsis-specific systemic inflammation classification.

**Figure 3:**
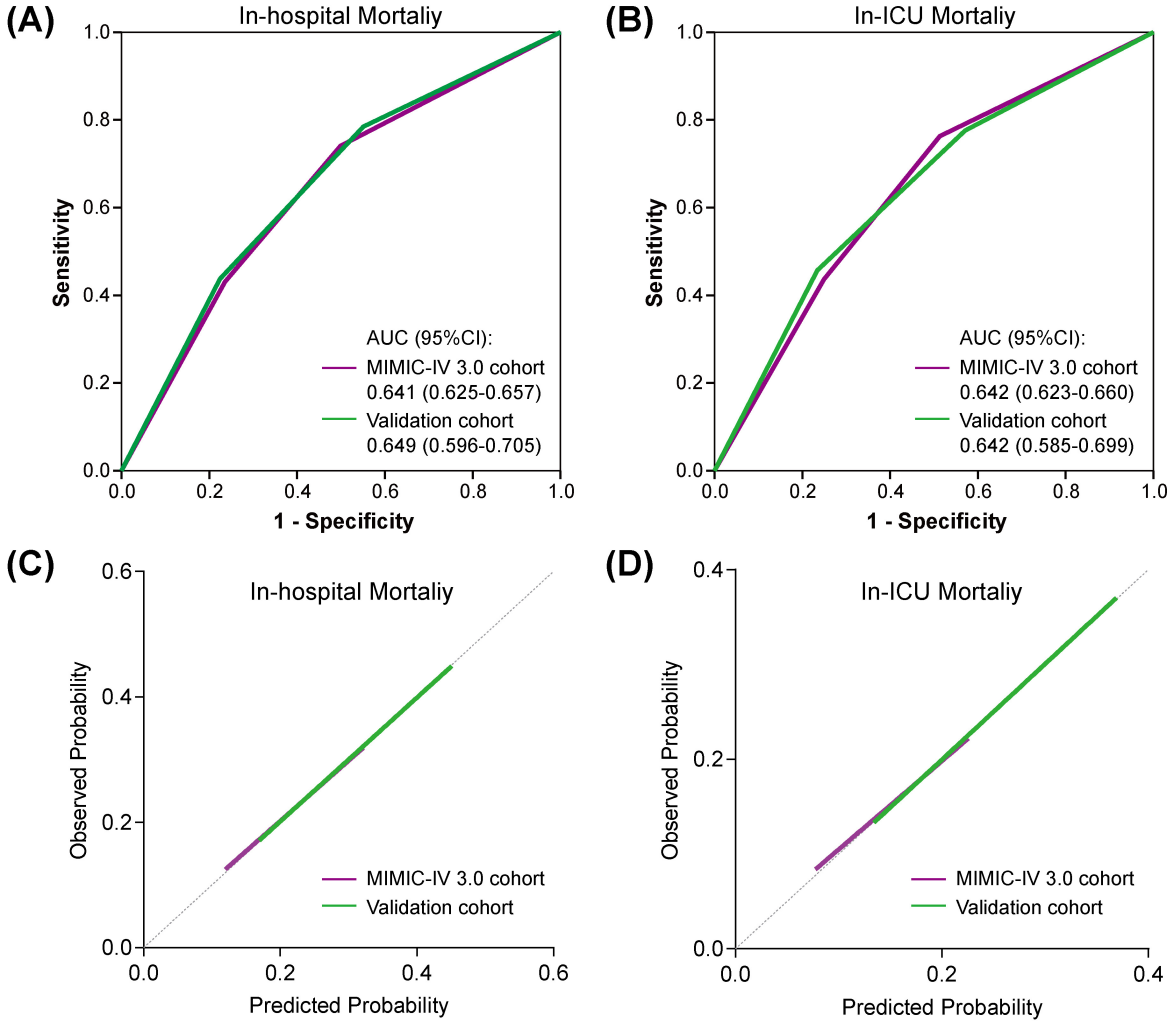
Predictive performance of the SSIC system for sepsis mortality in the MIMIC-IV 3.0 and validation cohorts. Receiver operating characteristic (ROC) curves **(A)** in-hospital mortality, **(B)** in-ICU mortality and calibration curves **(C)** in-hospital mortality, **(D)** in-ICU mortality are shown. AUC, area under the curve; CI, confidence interval; ICU, intensive care unit; SSIC, sepsis-specific systemic inflammation classification.

Using the SSIC system, systemic inflammation risk was assessed for 7,088 patients in the MIMIC-IV 3.0 cohort: 3,213 (45.3%) low, 1,931 (27.2%) moderate, and 1,944 (27.4%) high risk (Figure 2). In-hospital mortality incidences were 363 (11.3%), 434 (22.5%), and 603 (31.0%), respectively; in-ICU mortality incidences were 225 (7.0%), 310 (16.1%), and 414 (21.3%), respectively. The SSIC system showed moderate discriminative ability and good calibration for predicting both in-hospital and in-ICU mortality (Figure 3). Subgroup analysis showed that systemic inflammation risk identified by the SSIC system correlated with in-hospital and in-ICU mortality across almost all subgroups defined by age, gender, body mass index, comorbidities, severity scores, and interventions (Figure 2). Patients with high or moderate risk showed increased mortality risk compared to those with low risk.

#### 3.1.7 Combination of SSIC system and existing severity scores

**Figure 4:**
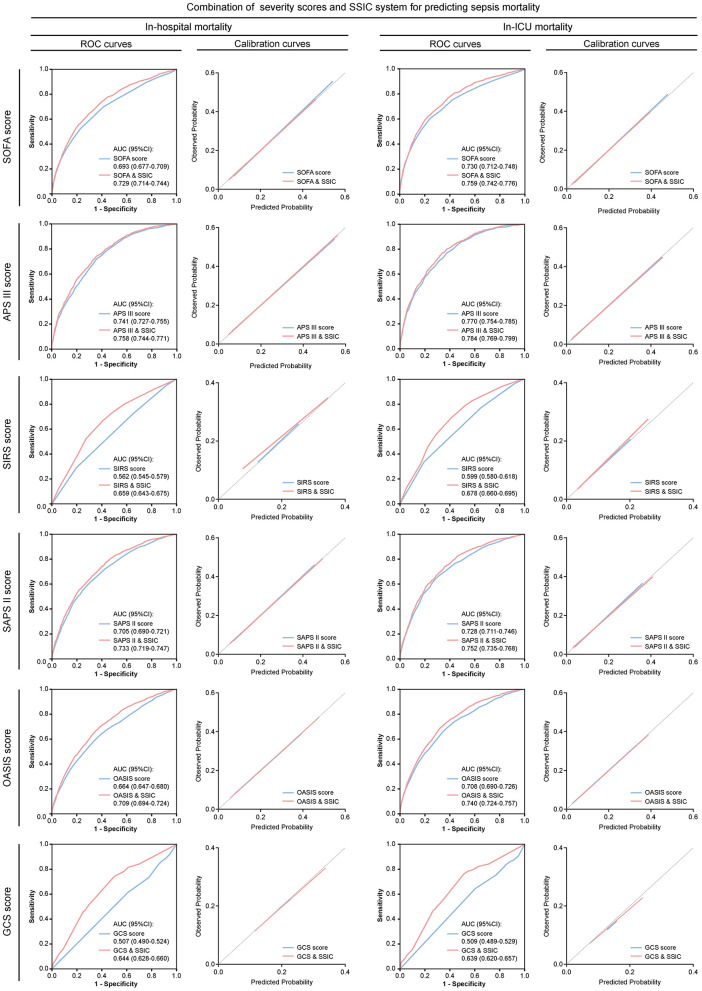
Improvement in predictive performance of the existing severity scores for sepsis mortality when combined with the SSIC system in the MIMIC-IV 3.0 Cohort. Receiver operating characteristic (ROC) curves were used to assess the discriminative ability. Calibration curves were created to illustrate the calibration. APS III, acute physiology score III; AUC, area under the curve; CI, confidence interval; GCS, Glasgow coma scale; ICU, intensive care unit; OASIS, Oxford acute severity of illness score; SAPS II, simplified acute physiology score II; SIRS, systemic inflammatory response syndrome; SOFA, sequential organ failure assessment; SSIC, sepsis-specific systemic inflammation classification.

As shown in Figure 3 and Figure 4, the SSIC system showed poorer discriminative ability than the SOFA score, APS III score, SAPS II score, and OASIS score in predicting in-hospital and in-ICU mortality, but greater performance than the SIRS score and GCS score. Regarding calibration for predicting sepsis mortality, the SSIC system and severity scores were comparably good. When combined with severity scores (Figure 4). The SSIC system improved the discriminative ability of all severity score for in-hospital and in-ICU mortality to different extents, with the largest improvement for the SIRS score and GCS score. However, the combination with SSIC system showed limited improvement in calibrations.

### 3.2 Institutional validation cohort

#### 3.2.1 Participants

The validation cohort sample size was determined based on MIMIC-IV 3.0 cohort outcomes. The in-hospital mortality rate was estimated at 30% for patients with high SSIC risk and 15% for patients with low-moderate SSIC risk. The sample sizes ratio between groups was estimated to be 1:2.5. With α = 0.05 and power = 0.95, the required sample size was at least 116 high SSIC risk and 290 low-moderate SSIC risk patients. We reviewed sepsis patients admitted to the ICU of the First Affiliated Hospital of Zhengzhou University between July 2023 and December 2024. Of 1,149 assessed patients, 653 were excluded, leaving 496 patients as the validation cohort (Figure 1). Mean age was 58.2 years (SD: 15.1), and 35.1% were female. In-hospital mortality occurred in 144 (29.0%) patients, and in-ICU mortality in 116 (23.4%).

#### 3.2.3 SSIC system and mortality risk

According to the SSIC system, systemic inflammation risk in the validation cohort was classified as low in 189 (38.1%), moderate in 165 (33.3%), and high in 142 (28.6%) patients. Characteristics by group are detailed in Table 5. No significant differences in demographics or severity scores were observed among risk groups. Mortality incidence rose with increasing SSIC risk: both in-hospital and in-ICU mortality rates were highest in the high-risk group, followed by the moderate- and low-risk groups.

**Table 5:** Characteristics of sepsis patients in the validation cohort (*N* = 496).

Characteristics	Systemic inflammation risk identified by the SSIC system			*P* value
	Low (*N* = 189)	Moderate (*N* = 165)	High (*N* = 142)	
*Demographic data*				
Age, years	58.3 ± 15.7	57.8 ± 14.5	58.3 ± 15.1	0.95
Gender (female)	63 (33.3)	62 (37.6)	49 (34.5)	0.70
Smoking history	50 (26.5)	44 (26.7)	36 (25.4)	0.90
Drinking history	34 (18)	35 (21.2)	34 (23.9)	0.53
Diabetes	67 (35.4)	55 (33.3)	38 (26.8)	0.23
Body mass index, kg/m^2^	23.4 (20.5–26.1)	22.9 (20.2–26.0)	24.0 (20.8–26.0)	0.50
NRS 2002 score	3 (1–4)	3 (1–4)	3 (1–3)	0.36
*Primary infection of sepsis*				
Respiratory system	78 (41.3)	81 (49.1)	66 (46.5)	0.025
Gastrointestinal tract	40 (21.2)	30 (18.2)	28 (19.7)	
Hepatobiliary system	9 (4.8)	12 (7.3)	18 (12.7)	
Nervous system	10 (5.3)	13 (7.9)	3 (2.1)	
Bone and skin soft tissue	22 (11.6)	14 (8.5)	11 (7.7)	
Urinary system	9 (4.8)	8 (4.8)	7 (4.9)	
Others	21 (11.1)	7 (4.2)	9 (6.3)	
*Severity scores*				
SOFA score	7 (5–11)	8 (5–12)	8 (5–11)	0.63
GCS score	13 (7–15)	15 (8–15)	15 (7–15)	0.25
*Systemic inflammation indicators*				
Serum glucose, mg/dL	130 (100–168)	160 (112–263)	189 (142–277)	<0.001
Total neutrophils, × 10^3^/μl	8.18 (5.28–11.9)	8.15 (4.17–11.6)	12.0 (8.72–16.5)	<0.001
Total lymphocytes, × 10^3^/μl	1.28 (0.91–1.85)	0.70 (0.47–0.95)	0.40 (0.26–0.62)	<0.001
NLR	4.33 (2.50–6.26)	8.15 (4.17–11.6)	19.6 (13.2–28.1)	<0.001
GLR	79.9 (52.5–113)	199 (141–317)	353 (229–591)	<0.001
*Outcomes*				
In-hospital mortality	31 (16.4)	50 (30.3)	63 (44.4)	<0.001
In-ICU mortality	26 (13.8)	37 (22.4)	53 (37.3)	<0.001

Data are mean ± standard deviation, number (percentage), or median (interquartile range). Differences among groups were assessed using ANOVA, Pearson's chi-squared tests, or Kruskal–Wallis tests. GCS, Glasgow coma scale; GLR, glucose-to-lymphocyte ratio; ICU, intensive care unit; NLR, neutrophil-to-lymphocyte ratio; NRS, Nutrition risk screening; SOFA, sequential organ failure assessment; SSIC, sepsis-specific systemic inflammation classification.

The SSIC system showed moderate discriminative ability and good calibration for predicting both in-hospital and in-ICU mortality in the validation cohort, which was comparable to that in the MIMIC-IV 3.0 cohort (Figure 2). Notably, the SSIC system consistently showed greater discriminative ability than NLR and GLR alone in predicting in-hospital mortality [AUC 95% CI: SSIC 0.649 (0.596–0.705); NLR 0.598 (0.541–0.655); GLR 0.590 (0.531–0.650)] and in-ICU mortality [AUC 95% CI: SSIC 0.642 (0.585–0.699); NLR 0.593 (0.537–0.659); GLR 0.594 (0.529–0.660)].

Multivariable analysis results are detailed in Table 6. After adjusting for age, gender, smoking history, comorbidities, body mass index, infection sites, SOFA score, and GCS score, high SSIC risk was independently associated with increased in-hospital and in-ICU mortality, while moderate risk was associated with increased in-hospital mortality and a non-significant increase in in-ICU mortality.

**Table 6:** Application of the SSIC system for predicting mortality in sepsis patients in the validation cohort (*N* = 496).

Endpoints	SSIC risk	Univariable analysis		Multivariable analysis [OR (95% CI)]			
		OR (95% CI)	*P* value	Model 1	*P* value	Model 2	*P* value
In-hospital mortality	High	4.07 (2.45–6.75)	**<0.001**	3.74 (2.24–6.24)	**<0.001**	2.91 (1.54–5.90)	**0.001**
	Moderate	2.22 (1.33–3.68)	**0.002**	2.08 (1.25–3.47)	**0.005**	1.61 (1.02–2.55)	**0.041**
	Low (reference)	–	–	–	–	–	–
In–ICU mortality	High	3.73 (2.19–6.38)	**<0.001**	3.44 (2.00–5.89)	**<0.001**	2.59 (1.30–5.17)	**0.007**
	Moderate	1.81 (1.04–3.15)	**0.035**	1.70 (0.98–2.97)	0.060	1.43 (0.92–2.22)	0.11
	Low (reference)	–	–	–	–	–	–

Multivariable analyses were conducted using logistic regression models with the backward conditional methods. The models varied based on the inclusion of different clinical characteristics as follows: Model 1 included adjustments for demographic data (age, gender, smoking history, comorbidities, and body mass index); Model 2 further incorporated adjustments for infection sites and severity assessment using the sequential organ failure assessment (SOFA) and Glasgow coma scale (GCS). CI, confidence interval; ICU, intensive care unit; OR, odds ratios; SSIC, sepsis-specific systemic inflammation classification. Bold values in the table indicate a statistically significant difference (*p* < 0.05).

## 4 Discussion

This study confirmed the association between nutrition and systemic inflammation status and sepsis mortality. Systemic inflammation indices generally showed greater predictive value for sepsis mortality than indices involving nutrition assessment. NLR and GLR demonstrated the best predictive performance. The SSIC system, which combines NLR and GLR, was established using the MIMIC-IV 3.0 cohort and validated in an independent institutional cohort for predicting mortality.

Sepsis is characterized by excessive systemic inflammation and immune imbalance, which inherently drive progressive systemic inflammatory activation and aggravated metabolic stress, forming a self-amplifying vicious cycle toward organ injury (1, 3). Therefore, systemic inflammation status has intrinsic potential for prognosticating sepsis mortality. Retrospective cohort studies have demonstrated associations between sepsis mortality and nutrition/inflammation-based indices such as NLR (7), GLR (16), SII (8), NPR (9), PLR (25), and SIRI (10). Our study confirmed the predictive value of these indices, and notably, was the first to confirm the predictive value of LCS. However, LMR (23), LCR (21), CAR (19), PNI (17), and GNRI (26), previously reported to be associated with sepsis mortality, showed no independently predictive value in our analysis. Additionally, AGR, COUNT, mGPS, SNII, ALI, and mGNRI were investigated for the first time in sepsis patients and were not independently associated with mortality. The significantly lower severity and mortality of patients excluded from the MIMIC-IV 3.0 cohort due to missing nutrition/inflammation data suggest that the missing data was more likely due to the absence of relevant laboratory tests. Laboratory tests for patients with lower severity and mortality risk may not have been ordered by physicians. These differences indicate that our findings, particularly the SSIC system, may be more suitable for sepsis patients with relatively severe conditions and high mortality risk. The external validation using an institutional cohort with a relatively high mortality rate further supports the SSIC system. The high rate of missing data for indices involving C-reactive proteins is a concern, which cannot be addressed by multiple imputation because of the missing rate exceeding 90% (35, 36).

Nutrition and systemic inflammation affect therapeutic outcomes in various diseases, but with varied performance across populations and endpoints. For example, ALI was optimal for predicting overall survival in lung cancer patients (4), while SNII (15) best predicted perioperative complications; neither was associated with sepsis mortality in this study. Similarly, PNI performed better than NLR for predicting survival in hemodialysis patients (37), whereas the reverse was true in our sepsis analysis. These findings highlight the importance of comparing different nutrition/inflammation-based indices and selecting optimal ones for assessing mortality risk in sepsis patients. Using ROC curves and multivariable analyses, through both indirect and direct comparisons, together with sensitive analysis in multiple imputation models, this study filled this knowledge gap, demonstrating that NLR and GLR have the best predictive performance. However, the relatively low AUC values of these indices indicate limited feasibility of their sole use in clinical decision-making. The SSIC system, combining NLR and GLR, was thus established to improve the predictive performance for sepsis mortality.

The system's predictive capability stems from its parameters. Peripheral lymphocyte counts reflect immune system integrity (38, 39), neutrophil counts indicate inflammation levels (40), and NLR captures the interplay between immune response and inflammation. Elevated serum glucose indicates metabolic stress from inflammation reactions, while increased glucagon, adrenaline, and cortisol secretion, and insulin resistance promote glucose mobilization (41, 42). Stress-induced hyperglycemia could also potentiate pro-inflammatory response of immune cells (41, 43). Thus, a high GLR indicates a state of high metabolism and inflammation, correlating with sepsis severity. Notably, the aggravation of systemic inflammatory burden and heightened metabolic stress are key pathophysiological characteristics of sepsis (1, 3). The SSIC system, which captures these pathophysiological parameters, can therefore be closely related to the prognosis of sepsis. These connections also explain why systemic inflammation indices show superiority over indices focused on nutrition assessment or combined nutrition and systemic inflammation in predicting sepsis mortality. However, given that hyperglycemia and leukocytosis are themselves components of sepsis severity, residual confounding by disease severity makes it difficult to fully disentangle the direct effect of these laboratory abnormalities from the underlying severity of the illness itself, which could potentially inflate the observed association between the SSIC system and sepsis mortality.

The application of the SSIC system should be considered in different populations and regions. In this study, the derivation cohort and validation cohort of the SSIC system have substantial differences in population characteristics and mortality risk. The MIMIC-IV 3.0 cohort consisted of patients admitted at the Beth Israel Deaconess Medical Center, while the validation cohort consisted of sepsis patients treated in an institution in China. These patients have different races, cultures, admission criteria, and treatment practices. The mortality rate of the validation cohort was also higher than that of the MIMIC-IV 3.0 cohort. The good performance of the SSIC system in the validation cohort for predicting sepsis mortality indicates its good application across different populations and institutions. However, the moderate SSIC risk was not associated with increased in-ICU mortality risk after adjustment in the validation cohort, which suggests the necessity of modifying the SSIC system to fit different populations. In general, the application of the SSIC system warrants testing and possible modification in different populations and regions.

Despite the consistent association between the SSIC system and sepsis mortality across different subpopulations, its discriminative ability was inferior to that of most existing severity scores, such as the SOFA, APS III, SAPS II, and OASIS scores. This limitation may stem from the system's sole focus on systemic inflammation rather than a comprehensive assessment of multiple organs and systems. The low HRs for moderate and high inflammation risk, as classified by the SSIC system in multivariable analysis, along with the moderate AUC values in ROC curve analysis, confirm their contribution to sepsis mortality but also indicate limited individual predictive accuracy. A previous study by Toscano et al. (44) also reported that nutrition/inflammation-based indices alone performed worse than systemic illness severity assessments for predicting in-hospital mortality in sepsis patients. Therefore, application of the SSIC system as part of a comprehensive assessment that includes clinical judgment and other relevant factors could be advisable. Our analysis also released the potential of the SSIC system to improve the discrimination ability for sepsis mortality when combined with severity scores. In clinical practice, systemic inflammation risk assessment using the SSIC system should be performed as early as possible—not limited to ICU admission but as soon as sepsis is suspected—to allow time for interventions. High-risk patients may benefit from targeted treatments, though the specific treatment approach warrants further investigation. The effectiveness of interventions to improve systemic inflammation status in reducing mortality risk also requires further study.

## Limitations

Several limitations of this study warrant acknowledgment. Despite the relatively large sample size of the MIMIC-IV 3.0 and validation cohorts, the retrospective design may introduce selection bias and limit generalizability. The comparisons between the included and excluded patients in the MIMIC-IV 3.0 database indicate that our findings are derived from sepsis patients with relatively high severity and mortality risk; their interpretation in milder patients warrants further investigation. Due to substantial missing data for several nutrition/inflammation-based indices, only ten of the 18 indices were direct compared; the remaining should be compared with well-documented indices in further studies. In multivariable analysis, despite adjusting for important clinical parameters, unmeasured or residual confounding may still persist. As the SSIC system was established solely based on limited biochemical parameters, its predictive accuracy for mortality is limited when used alone, and it is therefore recommended to be combined with existing severity scores for better risk assessment. Given the high missing rate of C-reactive protein, this marker was not incorporated into the SSIC system, and further research is therefore warranted. The application of the SSIC system for severity assessment and mortality prediction in sepsis patients requires prospective validation. The specific interventions guided by the SSIC system and their effectiveness in reducing mortality risk also require prospective investigation. Notably, moderate SSIC risk was not associated with increased in-ICU mortality after adjustment in the validation cohort, which should be considered in clinical use, and modifying the SSIC system to fit local populations may be helpful. Finally, dynamic changes in nutrition/inflammation-based indicators may also be related to sepsis mortality and should be investigated in future studies.

## Conclusions

This study confirms the potential of nutrition and systemic inflammation status for predicting mortality in sepsis patients. Comparison of 18 nutrition/inflammation-based indices provides benchmarks for future assessment tool development. NLR and GLR demonstrated the best predictive value among 18 indices, though discriminative ability was generally low. The SSIC system, which combines NLR and GLR, was established and validated to improve predictive ability for sepsis mortality. The SSIC system may be considered for further validation alongside existing severity scores for mortality prediction.

## Data Availability

The original contributions presented in the study are included in the article/Supplementary material, further inquiries can be directed to the corresponding authors.
