# Supplementary material for: Development and validation of a sepsis-specific systemic inflammation classification system for mortality prediction
Source: Front Med (Lausanne). 2026 Jun 26;13:1852863. doi: 10.3389/fmed.2026.1852863 (PMC13350257; doi:10.3389/fmed.2026.1852863)
Supplement: Supplementary file 1 [file Data_Sheet_1.PDF]

Development and validation of a sepsis-specific systemic inflammation classification system for mortality prediction.

Zhao Wu, Jianliang Cao, Qiang Zhang, Ruyi Lei, Yuepeng Hu, Shuguang Zhang, Chao Lan, Peiyu Wang, MD, Tongwen Sun, MD.

Supplementary Materials:

Supplementary Data 1: pg. 2

Supplementary Table 1: pg. 3-4

Supplementary Table 2: pg. 5

Supplementary Table 3: pg. 6

Supplementary Table 4: pg. 7

Supplementary Table 5: pg. 8

Supplementary Table 6: pg. 9

Supplementary Table 7: pg. 10-11

Supplementary Table 8: pg. 12

Supplementary Data 1

1. Sepsis definition

Patients were diagnosed with sepsis according to the Sepsis 3.0 diagnostic criteria. The critical criteria included:

(1) Clinical suspicion of infection as determined by the earlier timestamp of antibiotics administration and cultures within a certain timeframe. If antibiotics were given first, then the cultures must have been obtained within 24 hours. If cultures were obtained first, then antibiotics must have been subsequently ordered within 72 hours.

(2) The occurrence of end-organ damage as identified by a two-point deterioration in SOFA score.

(3) The onset time of sepsis is the earlier of time-suspicion and time-SOFA as long as time-SOFA occurs no more than 48 hours before or 24 hours after time-suspicion; otherwise, the patient is not marked as a sepsis patient. Specifically, if time-suspicion – 48h ≤ time-SOFA ≤ time-suspicion +24h, then time-sepsis = min (time-suspicion, time-SOFA).

2. SQL code

The Structured Query Language (SQL) code used for sepsis identification can be found at https://github.com/MIT-LCP/mimic-iv/tree/master/concepts/sepsis

Supplementary Table 1. Calculation of eighteen nutrition/inflammation-based indices.

Nutrition/inflammation-based indices	Abbreviations	Calculation methods
Albumin-to-globulin ratio	AGR	$\frac{\text{Serum albumin (g/L)}}{\text{Serum globulin (g/L)}}$
Neutrophil-to-lymphocyte ratio	NLR	$\frac{\text{Total neutrophils}}{\text{Total lymphocytes}}$
Platelet-to-lymphocyte ratio	PLR	$\frac{\text{Total platelets}}{\text{Total lymphocytes}}$
Neutrophil-to-platelet ratio	NPR	$\frac{\text{Total neutrophils}}{\text{Total platelets}}$
Lymphocyte-to-monocyte ratio	LMR	$\frac{\text{Total lymphocytes}}{\text{Total monocytes}}$
Systemic inflammation response index	SIRI	$\frac{\text{Total neutrophils} \times \text{Total monocytes}}{\text{Total lymphocytes}}$
Systemic immune-inflammation index	SII	$\frac{\text{Total neutrophils} \times \text{Total platelets}}{\text{Total lymphocytes}}$
Systemic nutrition-inflammation index	SNII	$\frac{\text{Total cholesterol (mmol/L)} \times \text{Total lymphocytes}}{\text{Total monocytes}}$
Prognostic nutritional index	PNI	$10 \times \text{Serum albumin (g/dL)} + 0.005 \times \text{Total lymphocytes (/µl)}$
Geriatric nutritional risk index	GNRI	$1.489 \times \text{Serum albumin (g/dL)} + 41.7 \times \frac{\text{present weight}}{\text{ideal weight}}$
Advanced lung cancer inflammation index	ALI	$\frac{\text{Body mass index } (\text{kg}/m^2) \times \text{Serum albumin (g/dL)}}{\text{(Total neutrophils/Total lymphocytes)}}$
Glucose-to-lymphocyte ratio	GLR	$\frac{\text{Serum glucose (mg/dL)}}{\text{Total lymphocytes } (\times 10^9/\text{L})}$
C-reactive protein-to-albumin ratio	CAR	$\frac{\text{Serum C reactive protein (mg/L)}}{\text{Serum albumin (g/L)}}$
Lymphocyte to C-reactive protein ratio	LCR	$\frac{10{,}000 \times \text{total lymphocytes } (\times 10^9/\text{L})}{\text{Serum C reactive protein (mg/L)}}$

Modified geriatric nutritional risk index	mGNRI	$\frac{14.89/\text{Serum C reactive protein (mg/L)}}{41.7 \times \text{(present weight/ideal weight)}}$
Modified Glasgow prognostic score	mGPS	Calculated based on levels of serum albumin and C-reactive protein
lymphocyte C-reactive protein score	LCS	Calculated based on levels of total lymphocytes and serum C-reactive protein
Controlling nutritional status score	COUNT	Calculated based on levels of serum albumin, total lymphocytes, and total cholesterol

Supplementary Table 2: Comparison of clinical characteristics between sepsis patients from the MIMIC-IV 3.0 database who were included in the study and those excluded due to missing data..

Characteristics	Included cohort (N = 11577)	Excluded cohort (N = 8297)	P value
Demographic data			
Age, years	60.4 ± 13.8	61.3 ± 13.9	<0.001
Gender (Female)	4523 (39.1)	3222 (38.8)	0.74
Body mass index, kg/m^2	28.7 (24.7-33.9)	29.0 (25.4-33.6)	0.032
Comorbidities			
Cardiovascular	7186 (62.1)	3432 (41.4)	<0.001
Liver	2082 (18.0)	804 (9.7)	<0.001
Renal	2214 (19.1)	1135 (13.7)	<0.001
Diabetes	3782 (32.7)	2654 (32.0)	0.31
COPD	1166 (10.1)	451 (5.4)	<0.001
Stroke	835 (7.2)	755 (9.1)	<0.001
Vital signs			
MAP, mmHg	79 (69-93)	77 (67-89)	<0.001
Heart rate, /min	89 (78-105)	86 (76-100)	<0.001
Respiratory rate, /min	19 (16-24)	17 (14-22)	<0.001
SpO_2, %	98 (95-100)	99 (96-100)	<0.001
Temperature, °C	36.8 (36.5-37.2)	36.8 (36.4-37.2)	<0.001
Severity scores			
SOFA score	6 (4-9)	4 (3-7)	<0.001
APS III score	49 (36-66)	40 (30-55)	<0.001
SIRS score	3 (2-3)	3 (2-3)	<0.001
SAPS II score	38 (30-48)	34 (27-42)	<0.001
OASIS score	34 (28-40)	32 (27-37)	<0.001
GCS score	15 (14-15)	15 (13-15)	<0.001
Interventions			
Mechanical ventilation	10427 (90.1)	7087 (85.4)	<0.001
Ventilation duration, hours	58.6 (21.3-143)	34.0 (19.8-67.1)	<0.001
CRRT	1421 (12.3)	213 (2.6)	<0.001
Outcomes			
In-hospital mortality	2281 (19.7)	822 (9.9)	<0.001
In-ICU mortality	1633 (14.1)	585 (7.1)	<0.001
Length of ICU stay, day	4.81 (2.34-10.0)	2.30 (1.47-4.02)	<0.001
Length of hospital stay, day	12.9 (7.18-22.8)	7.05 (4.64-11.5)	<0.001

Data are mean ± standard deviation, number (percentage), or median (interquartile range).
Differences between groups were assessed using ANOVA, Pearson's chi-squared tests, or Kruskal–Wallis tests.
APS III: acute physiology score III; COPD: chronic obstructive pulmonary disease; CRRT: continuous renal replacement therapy; GCS: Glasgow coma scale; ICU: intensive care unit; MAP: mean arterial pressure; OASIS: Oxford acute severity of illness score; SAPS II: simplified acute physiology score II; SIRS: systemic inflammatory response syndrome; SOFA: sequential organ failure assessment; SpO_2: peripheral capillary oxygen saturation.

Supplementary Table 3: Values of nutrition/inflammation-based indices in the MIMIC-IV 3.0 cohort before and after multiple imputation for missing data.

Indices	Original data	Multiple imputations (N = 11577)				
		Imputation 1	Imputation 2	Imputation 3	Imputation 4	Imputation 5
NLR	7.94 (4.39-15.2)	7.77 (4.14-15.6)	7.77 (4.16-15.7)	7.93 (4.21-15.9)	7.86 (4.18-15.9)	7.76 (4.14-15.7)
PLR, ×10	15.1 (8.18-27.5)	15.0 (7.58-28.3)	15.1 (7.59-28.05)	15.2 (7.68-28.1)	15.2 (7.70-28.3)	14.9 (7.56-27.9)
NPR, $\times 10^{-2}$	5.59 (3.44-8.79)	5.58 (3.49-9.15)	5.64 (3.51-9.09)	5.64 (3.5-9.16)	5.71 (3.51-9.18)	5.61 (3.48-9.16)
LMR	1.72 (0.93-3.46)	1.68 (0.81-3.39)	1.67 (0.80-3.38)	1.66 (0.8-3.37)	1.71 (0.83-3.42)	1.70 (0.82-3.42)
SIRI	5.40 (2.08-13.4)	5.43 (1.45-13.6)	5.50 (1.53-13.5)	5.50 (1.51-13.7)	5.22 (1.32-13.3)	5.49 (1.46-13.4)
SII, $\times 10^{2}$	13.3 (6.26-28.9)	13.3 (5.91-28.5)	13.4 (5.84-27.8)	13.6 (5.92-28.4)	13. 2(5.84-28.2)	13.14 (5.90-27.8)
PNI	32.2 (20.8-38.9)	31.0 (21.1-39.9)	30.8 (20.7-39.5)	31.0 (21.1-40.0)	30.9 (20.7-39.6)	31.0 (21.0-39.8)
GNRI	99.3 (88.5-112)	99.3 (88.2-111)	99.6 (88.2-112)	99.3 (88.1-112)	99.2 (87.9-111.1)	99.6 (88.2-115)
ALI	9.42 (4.53-17.7)	9.37 (2.82-21.6)	9.11 (2.78-21.1)	9.13 (2.72-21.0)	9.06 (2.83-21.4)	9.35 (2.72-21.3)
GLR, $\times 10^{2}$	1.24 (0.71-2.30)	1.25 (0.65-2.96)	1.27 (0.65-3.06)	1.25 (0.65-3.00)	1.27 (0.65-3.08)	1.28 (0.65-3.02)

Data are medians (interquartile ranges).

Markov Chain Monte Carlo (MCMC) multiple imputation methods are used to impute the missing values of nutrition/inflammation-based indices. Five imputations were established and reported.

ALI: advanced lung cancer inflammation index; GLR: glucose-to-lymphocyte ratio; GNRI: geriatric nutritional risk index; LMR: lymphocyte-to-monocyte ratio; NLR: neutrophil-to-lymphocyte ratio; NPR: neutrophil-to-platelet ratio; PLR: platelet-to-lymphocyte ratio; PNI: prognostic nutritional index; SII: systemic immune-inflammation index; SIRI: systemic inflammation response index.

Supplementary Table 4: Analysis of predictive factors for in-hospital mortality in sepsis patients in the MIMIC-IV 3.0 cohort.

Characteristics	Comparisons	Logistic regression models				Cox proportional hazards regression models			
		Univariable analysis		Multivariable analysis [1]		Univariable analysis		Multivariable analysis [1]	
		OR (95% CI)	*P* value	OR (95% CI)	*P* value	HR (95% CI)	*P* value	HR (95% CI)	*P* value
Demographic data									
Age, years	Per 1 year	1.013 (1.010-1.017)	<0.001	1.020 (1.015-1.024)	<0.001	1.015 (1.012-1.019)	<0.001	1.020 (1.016-1.024)	<0.001
Gender (Female)	Female vs. male	1.10 (1.00-1.20)	0.056			1.06 (0.98-1.16)	0.15		
Body mass index	Per 1 kg/m^2	1.003 (0.997-1.008)	0.41			1.001 (0.996-1.006)	0.67		
Comorbidities									
Cardiovascular	Yes vs. No	3.55 (3.16-3.99)	<0.001	1.64 (1.44-1.87)	<0.001	2.39 (2.14-2.66)	<0.001	1.36 (1.20-1.53)	<0.001
Liver	Yes vs. No	2.06 (1.85-2.29)	<0.001	1.54 (1.36-1.75)	<0.001	1.57 (1.43-1.72)	<0.001	1.31 (1.18-1.45)	<0.001
Renal	Yes vs. No	1.46 (1.31-1.63)	<0.001			1.25 (1.14-1.38)	<0.001	0.89 (0.81-0.99)	0.033
Diabetes	Yes vs. No	1.10 (0.99-1.21)	0.067			1.06 (0.97-1.16)	0.17		
COPD	Yes vs. No	1.42 (1.24-1.64)	<0.001	1.27 (1.08-1.49)	0.003	1.35 (1.19-1.53)	<0.001	1.27 (1.11-1.45)	<0.001
Stroke	Yes vs. No	1.00 (0.84-1.20)	0.97			1.01 (0.86-1.18)	0.93		
Vital signs									
Mean arterial pressure	Pear 10 mmHg	0.98 (0.96-1.01)	0.11			0.97 (0.94-0.99)	0.002		
Heart rate	Pear 10/min	1.09 (1.07-1.11)	<0.001			1.04 (1.02-1.06)	<0.001		
Respiratory rate	Pear 1/min	1.04 (1.04-1.05)	<0.001	1.022 (1.014-1.030)	<0.001	1.02 (1.02-1.03)	<0.001	1.010 (1.004-1.016)	0.002
SpO_2	Pear 5%	0.77 (0.73-0.81)	<0.001	0.89 (0.84-0.94)	<0.001	0.85 (0.82-0.88)	<0.001	0.92 (0.88-0.96)	<0.001
Temperature	Pear 1℃	0.97 (0.95-0.98)	<0.001	0.97 (0.95-0.99)	0.012	0.98 (0.97-0.99)	0.001		
Severity scores									
SOFA score	Per 1 score	1.20 (1.18-1.21)	<0.001			1.13 (1.12-1.14)	<0.001	1.02 (1.01-1.04)	0.011
APS III score	Per 5 score	1.037 (1.035-1.039)	<0.001	1.033 (1.030-1.037)	<0.001	1.037 (1.035-1.039)	<0.001	1.021 (1.018-1.024)	<0.001
SIRS score	Per 1 score	1.30 (1.23-1.37)	<0.001			1.22 (1.16-1.28)	<0.001		
SAPS II score	Per 1 score	1.052 (1.048-1.055)	<0.001			1.034 (1.032-1.037)	<0.001		
OASIS score	Per 1 score	1.073 (1.067-1.079)	<0.001	1.008 (1.000-1.016)	0.041	1.051 (1.046-1.056)	<0.001		
GCS score	Per 1 score	0.97 (0.96-0.98)	<0.001	0.96 (0.94-0.98)	<0.001	0.98 (0.97-0.99)	0.003		
Nutrition/inflammation-based indicators [2]									
Nutrition/inflammation-based items [2]									
Serum albumin	Per 1 g/L	0.71 (0.66-0.76)	<0.001	0.86 (0.79-0.94)	<0.001	0.88 (0.83-0.94)	<0.001		
Serum globulin	Per 1 g/L	0.72 (0.56-0.92)	0.009	0.71 (0.55-0.91)	0.007	0.81 (0.66-0.99)	0.042		
C-reactive protein	Per 1 mg/L	1.001 (0.999-1.003)	0.26	-		1.000 (0.999-1.002)	0.62		
Total cholesterol	Per 1 mg/dL	0.998 (0.996-1.001)	0.14	-		0.999 (0.997-1.001)	0.61		
Serum glucose	Per 1 mg/dL	1.002 (1.001-1.002)	<0.001	-		1.002 (1.001-1.002)	<0.001	1.001 (1.000-1.001)	0.003
Hemoglobin	Per 1 g/dL	0.96 (0.94-0.98)	<0.001	-		0.99 (0.97-1.00)	0.078		
Total neutrophils	Per 1×10^3/μl	1.039 (1.031-1.046)	<0.001	1.014 (1.006-1.023)	0.001	1.026 (1.020-1.032)	<0.001	1.012 (1.006-1.018)	<0.001
Total lymphocytes	Per 1×10^3/μl	1.001 (0.990-1.012)	0.81			1.002 (0.994-1.010)	0.58		
Total monocytes	Per 1×10^3/μl	1.18 (1.09-1.29)	<0.001			1.05 (1.01-1.09)	0.017		
Total platelet	Per 1×10^4/μl	0.990 (0.986-0.994)	<0.001			0.991 (0.987-0.994)	<0.001		
Nutrition/inflammation-based indices [2]									
AGR	Per 1 unit	0.98 (0.87-1.11)	0.78			0.96 (0.87-1.07)	0.49		
NLR	Per 1 unit	1.020 (1.017-1.023)	<0.001	1.012 (1.009-1.015)	<0.001	1.010 (1.008-1.011)	<0.001	1.008 (1.006-1.010)	<0.001
PLR	Per 10 unit	1.005 (1.003-1.006)	<0.001	1.004 (1.002-1.005)	<0.001	1.002 (1.001-1.003)	<0.001	1.002 (1.001-1.003)	<0.001
NPR	Per 0.01 unit	1.034 (1.027-1.041)	<0.001	1.012 (1.006-1.018)	<0.001	1.012 (1.010-1.014)	<0.001	1.007 (1.004-1.011)	<0.001
LMR	Per 1 unit	0.966 (0.947-0.985)	<0.001			0.995 (0.982-1.009)	0.48		
SIRI	Per 1 unit	1.014 (1.011-1.018)	<0.001	1.006 (1.003-1.010)	<0.001	1.007 (1.005-1.008)	<0.001	1.004 (1.002-1.006)	<0.001
SII	Per 100 unit	1.006 (1.005-1.007)	<0.001	1.004 (1.003-1.005)	<0.001	1.003 (1.003-1.004)	<0.001	1.003 (1.002-1.004)	<0.001
SNII	Per 10 unit	1.000 (0.998-1.003)	0.63			1.001 (0.999-1.002)	0.49		
PNI	Per 1 unit	1.005 (1.002-1.009)	0.001			1.001 (0.999-1.002)	0.41		
GNRI	Per 1 unit	0.995 (0.992-0.998)	0.001	0.993 (0.990-0.996)	<0.001	0.998 (0.996-1.001)	0.19		
ALI	Per 1 unit	1.000 (1.000-1.000)	0.58			1.000 (1.000-1.000)	0.73		
GLR	Per 100 unit	1.053 (1.039-1.068)	<0.001	1.029 (1.016-1.042)	<0.001	1.007 (1.004-1.010)	<0.001	1.005 (1.002-1.008)	0.002
CAR	Per 10 unit	1.039 (0.989-1.091)	0.13			1.023 (0.980-1.068)	0.30		
LCR	Per 1 unit	1.000 (1.000-1.000)	0.79			1.000 (1.000-1.000)	0.25		
mGNRI	Per 1 unit	1.009 (0.997-1.021)	0.16			1.007 (0.997-1.017)	0.18		
mGPS	Per 1 score	1.62 (1.13-2.34)	0.009			1.39 (1.01-1.93)	0.046		
LCS	Per 1 score	2.49 (1.65-3.75)	<0.001	2.40 (1.49-3.88)	<0.001	1.92 (1.34-2.75)	<0.001	2.02 (1.39-2.93)	0.004
COUNT	Per 1 score	1.07 (1.02-1.13)	0.010			1.00 (0.96-1.05)	0.87		

[1] Multivariable analyses were conducted using logistic regression models or Cox proportional hazards regression models with the backward conditional methods. Results are presented as odds ratios (ORs) or hazard ratios (HRs) with 95% confidence intervals (CIs). Variables with a *P*-value of less than 0.10 in univariate analysis were selected for inclusion in multivariate regression models.

[2] Nutrition/inflammation-based indicators were individually incorporated into multivariate analyses. Clinical characteristics independently associated with mortality were selected as adjustment factors.

AGR: albumin-to-globulin ratio; ALI: advanced lung cancer inflammation index; APS III: acute physiology score III; CAR: C-reactive protein-to-albumin ratio; COPD: chronic obstructive pulmonary disease; CONUT: controlling nutritional status score; GCS: Glasgow coma scale; GLR: glucose-to-lymphocyte ratio; GNRI: geriatric nutritional risk index; LCR: lymphocyte to C-reactive protein ratio; LCS, lymphocyte C-reactive protein score; LMR: lymphocyte-to-monocyte ratio; MAP: mean arterial pressure; mGNRI: modified geriatric nutritional risk index; mGPS: modified Glasgow prognostic score; NLR: neutrophil-to-lymphocyte ratio; NPR: neutrophil-to-platelet ratio; OASIS: Oxford acute severity of illness score; PLR: platelet-to-lymphocyte ratio; PNI: prognostic nutritional index; SAPS II: simplified acute physiology score II; SII: systemic immune-inflammation index; SIRI: systemic inflammation response index; SIRS: systemic inflammatory response syndrome; SNII: systemic nutrition-inflammation index; SOFA: sequential organ failure assessment; SpO_2: peripheral capillary oxygen saturation.

Supplementary Table 5: Analysis of predictive factors for in-ICU mortality in sepsis patients in the MIMIC-IV 3.0 cohort.

Characteristics	Comparisons	Logistic regression models				Cox proportional hazards regression models			
		Univariable analysis		Multivariable analysis [1]		Univariable analysis		Multivariable analysis [1]	
		OR (95% CI)	*P* value	OR (95% CI)	*P* value	HR (95% CI)	*P* value	HR (95% CI)	*P* value
Demographic data									
Age, years	Per 1 year	1.009 (1.005-1.013)	<0.001	1.014 (1.008-1.019)	<0.001	1.013 (1.010-1.017)	<0.001	1.020 (1.016-1.025)	<0.001
Gender (Female)	Female vs. male	1.07 (0.96-1.19)	0.23			1.07 (0.97-1.18)	0.18		
Body mass index	Per 1 kg/m^2	1.009 (1.003-1.015)	0.005			0.998 (0.993-1.004)	0.59		
Comorbidities									
Cardiovascular	Yes vs. No	3.36 (2.94-3.84)	<0.001	1.32 (1.13-1.55)	0.001	2.37 (2.09-2.70)	<0.001	1.37 (1.19-1.59)	<0.001
Liver	Yes vs. No	1.68 (1.48-1.90)	<0.001	1.15 (0.99-1.34)	0.065	1.84 (1.65-2.06)	<0.001	1.57 (1.38-1.78)	<0.001
Renal	Yes vs. No	1.34 (1.18-1.52)	<0.001			1.35 (1.21-1.52)	<0.001		
Diabetes	Yes vs. No	1.02 (0.91-1.13)	0.80			1.05 (0.95-1.17)	0.34		
COPD	Yes vs. No	1.36 (1.16-1.60)	<0.001	1.28 (1.07-1.55)	0.009	1.27 (1.09-1.46)	0.002	1.20 (1.03-1.41)	0.022
Stroke	Yes vs. No	0.92 (0.75-1.13)	0.42			0.88 (0.73-1.07)	0.23		
Vital signs									
MAP	Pear 10 mmHg	0.98 (0.95-1.01)	0.16			0.95 (0.93-0.98)	<0.001		
Heart rate	Pear 10/min	1.09 (1.07-1.12)	<0.001			1.05 (1.03-1.08)	<0.001		
Respiratory rate	Pear 1/min	1.043 (1.035-1.050)	<0.001	1.016 (1.008-1.025)	<0.001	1.021 (1.014-1.028)	<0.001	1.011 (1.003-1.019)	0.005
SpO_2	Pear 5%	0.74 (0.71-0.78)	<0.001	0.88 (0.83-0.94)	<0.001	0.87 (0.84-0.91)	<0.001	0.95 (0.90-0.99)	0.026
Temperature	Pear 1°C	0.971 (0.953-0.990)	0.003			0.984 (0.969-0.999)	0.038		
Severity scores									
SOFA score	Per 1 score	1.23 (1.21-1.24)	<0.001			1.14 (1.13-1.15)	<0.001	1.03 (1.02-1.05)	<0.001
APS III score	Per 5 score	1.040 (1.038-1.043)	<0.001	1.034 (1.030-1.039)	<0.001	1.022 (1.021-1.024)	<0.001	1.015 (1.012-1.018)	<0.001
SIRS score	Per 1 score	1.50 (1.40-1.60)	<0.001	1.12 (1.03-1.21)	0.006	1.28 (1.20-1.35)	<0.001	1.12 (1.05-1.19)	0.001
SAPS II score	Per 1 score	1.056 (1.052-1.060)	<0.001			1.034 (1.031-1.037)	<0.001		
OASIS score	Per 1 score	1.093 (1.086-1.100)	<0.001	1.023 (1.014-1.033)	<0.001	1.050 (1.044-1.056)	<0.001		
GCS score	Per 1 score	0.97 (0.96-0.98)	<0.001			0.99 (0.98-1.00)	0.089		
Nutrition/inflammation-based indicators [2]									
Nutrition/inflammation-based items [2]									
Serum albumin	Per 1 g/L	0.73 (0.67-0.80)	<0.001	-		0.88 (0.81-0.95)	0.001		
Serum globulin	Per 1 g/L	0.82 (0.61-1.11)	0.19	-		0.81 (0.61-1.07)	0.13		
C-reactive protein	Per 1 mg/L	1.002 (0.999-1.004)	0.13	-		1.001 (0.999-1.003)	0.40		
Total cholesterol	Per 1 mg/dL	1.000 (0.997-1.002)	0.80	-		0.999 (0.996-1.001)	0.31		
Serum glucose	Per 1 mg/dL	1.002 (1.002-1.003)	<0.001	1.001 (1.000-1.001)	0.004	1.001 (1.001-1.002)	<0.001	1.001 (1.000-1.001)	0.002
Hemoglobin	Per 1 g/dL	1.008 (0.986-1.030)	0.48	-		0.956 (0.937-0.975)	<0.001		
Total neutrophils	Per 1×10^3/μl	1.043 (1.035-1.051)	<0.001	1.012 (1.003-1.021)	0.013	1.028 (1.021-1.036)	<0.001	1.010 (1.003-1.017)	0.007
Total lymphocytes	Per 1×10^3/μl	1.005 (0.995-1.015)	0.34			1.002 (0.995-1.009)	0.66		
Total monocytes	Per 1×10^3/μl	1.08 (1.01-1.16)	0.029			1.04 (0.98-1.11)	0.16		
Total platelet	Per 1×10^4/μl	0.994 (0.989-0.999)	0.011			0.986 (0.981-0.990)	<0.001		
Nutrition/inflammation-based indices [2]									
AGR	Per 1 unit	1.06 (0.93-1.20)	0.43			1.00 (0.91-1.11)	0.94		
NLR	Per 1 unit	1.017 (1.014-1.021)	<0.001	1.009 (1.005-1.012)	<0.001	1.010 (1.008-1.012)	<0.001	1.006 (1.003-1.009)	<0.001
PLR	Per 10 unit	1.004 (1.003-1.006)	<0.001	1.003 (1.002-1.005)	<0.001	1.002 (1.001-1.003)	<0.001	1.002 (1.001-1.003)	<0.001
NPR	Per 0.01 unit	1.025 (1.019-1.032)	<0.001			1.013 (1.010-1.015)	<0.001	1.006 (1.001-1.010)	0.012
LMR	Per 1 unit	0.978 (0.958-0.998)	0.031			1.004 (0.994-1.014)	0.45		
SIRI	Per 1 unit	1.010 (1.007-1.013)	<0.001	1.004 (1.000-1.007)	0.034	1.006 (1.004-1.008)	<0.001		
SII	Per 100 unit	1.005 (1.004-1.007)	<0.001	1.003 (1.002-1.005)	<0.001	1.003 (1.002-1.004)	<0.001	1.003 (1.001-1.004)	<0.001
SNII	Per 10 unit	1.001 (0.999-1.003)	0.56	-		1.001 (0.999-1.003)	0.47		
PNI	Per 1 unit	1.003 (1.001-1.005)	0.007	-		1.000 (0.998-1.002)	0.84		
GNRI	Per 1 unit	0.999 (0.995-1.002)	0.34	-		0.998 (0.995-1.000)	0.073		
ALI	Per 1 unit	1.000 (1.000-1.000)	0.41			1.000 (1.000-1.000)	0.74		
GLR	Per 100 unit	1.046 (1.033-1.060)	<0.001	1.027 (1.014-1.041)	<0.001	1.007 (1.004-1.010)	<0.001	1.006 (1.002-1.009)	0.001
CAR	Per 10 unit	1.048 (0.992-1.107)	0.095			1.047 (0.992-1.105)	0.093		
LCR	Per 1 unit	1.000 (1.000-1.000)	0.28			1.000 (1.000-1.000)	0.23		
mGNRI	Per 1 unit	1.012 (0.999-1.025)	0.065			1.006 (0.994-1.018)	0.30		
mGPS	Per 1 score	1.48 (0.98-2.24)	0.065			1.51 (1.03-2.23)	0.036		
LCS	Per 1 score	2.25 (1.42-3.56)	0.001	2.14 (1.24-3.69)	0.006	2.01 (1.32-3.06)	0.001	1.98 (1.26-3.09)	0.003
COUNT	Per 1 score	1.05 (0.98-1.12)	0.17			1.01 (0.95-1.07)	0.79		

[1] Multivariable analyses were conducted using logistic regression models or Cox proportional hazards regression models with the backward conditional methods. Results are presented as odds ratios (ORs) or hazard ratios (HRs) with 95% confidence intervals (CIs). Variables with a *P*-value of less than 0.10 in univariate analysis were selected for inclusion in multivariate regression models.

[2] Nutrition/inflammation-based indicators were individually incorporated into multivariate analyses. Clinical characteristics independently associated with mortality were selected as adjustment factors.

AGR: albumin-to-globulin ratio; ALI: advanced lung cancer inflammation index; APS III: acute physiology score III; CAR: C-reactive protein-to-albumin ratio; COPD: chronic obstructive pulmonary disease; CONUT: controlling nutritional status score; GCS: Glasgow coma scale; GLR: glucose-to-lymphocyte ratio; GNRI: geriatric nutritional risk index; ICU: intensive care unit; LCR: lymphocyte to C-reactive protein ratio; LCS, lymphocyte C-reactive protein score; LMR: lymphocyte-to-monocyte ratio; MAP: mean arterial pressure; mGNRI: modified geriatric nutritional risk index; mGPS: modified Glasgow prognostic score; NLR: neutrophil-to-lymphocyte ratio; NPR: neutrophil-to-platelet ratio; OASIS: Oxford acute severity of illness score; PLR: platelet-to-lymphocyte ratio; PNI: prognostic nutritional index; SAPS II: simplified acute physiology score II; SII: systemic immune-inflammation index; SIRI: systemic inflammation response index; SIRS: systemic inflammatory response syndrome; SNII: systemic nutrition-inflammation index; SOFA: sequential organ failure assessment; SpO_2: peripheral capillary oxygen saturation.

Supplementary Table 6: Collinearity analysis of parameters included in the multivariable logistic regression models or Cox proportional hazards regression models.

Parameters	Collinearity statistics	
	Tolerance	Variance Inflation Factor
Demographic data		
Age, years	0.639	1.564
Comorbidities		
Cardiovascular	0.776	1.289
Liver	0.845	1.183
Renal	0.877	1.140
COPD	0.955	1.047
Vital signs		
Respiratory rate, /min	0.905	1.105
SpO_2, %	0.999	1.001
Temperature, °C	0.993	1.007
Severity scores		
SOFA score	0.408	2.452
APS III score	0.267	3.748
SIRS score	0.844	1.185
OASIS score	0.484	2.065
GCS score	0.631	1.585

APS III: acute physiology score III; COPD: chronic obstructive pulmonary disease; CRRT: continuous renal replacement therapy; GCS: Glasgow coma scale; OASIS: Oxford acute severity of illness score; SIRS: systemic inflammatory response syndrome; SOFA: sequential organ failure assessment; SpO_2: peripheral capillary oxygen saturation.

Supplementary Table 7: Discriminative ability of ten nutrition/inflammation-based indices for sepsis mortality in the MIMIC-IV 3.0 cohort after multiple imputation (*N* = 11577).

Imputations	In-hospital mortality		In-ICU mortality		Association
	AUC (95% CI)	*P* value	AUC (95% CI)	*P* value	
Imputation 1					
NLR	0.583 (0.570-0.596)	0.007	0.585 (0.571-0.600)	0.007	Positive
PLR	0.542 (0.529-0.555)	0.007	0.549 (0.534-0.564)	0.008	Positive
NPR	0.552 (0.538-0.565)	0.007	0.557 (0.541-0.572)	0.008	Positive
LMR	0.541 (0.527-0.554)	0.007	0.529 (0.513-0.545)	0.008	Negative
SIRI	0.560 (0.546-0.573)	0.007	0.563 (0.548-0.579)	0.008	Positive
SII	0.552 (0.539-0.565)	0.007	0.562 (0.547-0.577)	0.008	Positive
PNI	0.510 (0.497-0.523)	0.007	0.509 (0.494-0.524)	0.008	Positive
GNRI	0.520 (0.506-0.533)	0.007	0.510 (0.495-0.526)	0.008	Negative
ALI	0.564 (0.551-0.576)	0.007	0.573 (0.559-0.588)	0.008	Negative
GLR	0.577 (0.564-0.591)	0.006	0.586 (0.570-0.601)	0.007	Positive
Imputation 2					
NLR	0.591 (0.579-0.604)	0.006	0.601 (0.587-0.616)	0.007	Positive
PLR	0.539 (0.526-0.553)	0.007	0.546 (0.531-0.562)	0.008	Positive
NPR	0.558 (0.544-0.571)	0.007	0.567 (0.552-0.583)	0.008	Positive
LMR	0.542 (0.528-0.556)	0.007	0.536 (0.521-0.552)	0.008	Negative
SIRI	0.550 (0.536-0.563)	0.007	0.551 (0.536-0.567)	0.008	Positive
SII	0.554 (0.541-0.568)	0.007	0.566 (0.551-0.581)	0.008	Positive
PNI	0.507 (0.494-0.520)	0.007	0.506 (0.491-0.521)	0.008	Positive
GNRI	0.521 (0.507-0.535)	0.007	0.513 (0.498-0.529)	0.008	Negative
ALI	0.569 (0.556-0.581)	0.007	0.577 (0.561-0.593)	0.008	Negative
GLR	0.576 (0.562-0.590)	0.006	0.580 (0.566-0.594)	0.007	Positive
Imputation 3					
NLR	0.585 (0.573-0.598)	0.007	0.592 (0.578-0.607)	0.007	Positive
PLR	0.534 (0.521-0.548)	0.007	0.540 (0.524-0.555)	0.008	Positive
NPR	0.563 (0.550-0.576)	0.007	0.572 (0.556-0.587)	0.008	Positive
LMR	0.553 (0.539-0.566)	0.007	0.545 (0.530-0.561)	0.008	Negative
SIRI	0.555 (0.541-0.568)	0.007	0.558 (0.542-0.573)	0.008	Positive
SII	0.552 (0.539-0.566)	0.007	0.562 (0.547-0.577)	0.008	Positive
PNI	0.507 (0.494-0.519)	0.007	0.509 (0.494-0.524)	0.008	Positive
GNRI	0.524 (0.510-0.538)	0.007	0.512 (0.496-0.527)	0.008	Negative
ALI	0.568 (0.556-0.581)	0.007	0.577 (0.561-0.593)	0.008	Negative
GLR	0.571 (0.557-0.585)	0.006	0.579 (0.564-0.593)	0.007	Positive
Imputation 4					
NLR	0.588 (0.575-0.601)	0.007	0.594 (0.579-0.608)	0.007	Positive
PLR	0.542 (0.528-0.555)	0.007	0.551 (0.535-0.566)	0.008	Positive
NPR	0.559 (0.546-0.573)	0.007	0.566 (0.550-0.581)	0.008	Positive
LMR	0.555 (0.542-0.569)	0.007	0.537 (0.522-0.553)	0.008	Negative
SIRI	0.571 (0.557-0.584)	0.007	0.577 (0.562-0.592)	0.008	Positive
SII	0.560 (0.546-0.573)	0.007	0.568 (0.554-0.583)	0.008	Positive
PNI	0.509 (0.497-0.522)	0.007	0.515 (0.500-0.530)	0.008	Positive
GNRI	0.522 (0.508-0.535)	0.007	0.514 (0.498-0.529)	0.008	Negative
ALI	0.565 (0.552-0.578)	0.007	0.576 (0.560-0.592)	0.008	Negative
GLR	0.576 (0.562-0.590)	0.007	0.576 (0.561-0.590)	0.007	Positive
Imputation 5					

NLR	0.585 (0.572-0.597)	0.007	0.592 (0.578-0.606)	0.007
PLR	0.536 (0.522-0.549)	0.007	0.545 (0.529-0.560)	0.008
NPR	0.558 (0.544-0.571)	0.007	0.563 (0.547-0.578)	0.008
LMR	0.536 (0.522-0.550)	0.007	0.527 (0.511-0.542)	0.008
SIRI	0.559 (0.545-0.572)	0.007	0.561 (0.546-0.577)	0.008
SII	0.552 (0.539-0.566)	0.007	0.564 (0.549-0.579)	0.008
PNI	0.515 (0.502-0.528)	0.007	0.515 (0.500-0.530)	0.008
GNRI	0.526 (0.513-0.540)	0.007	0.516 (0.500-0.531)	0.008
ALI	0.567 (0.553-0.581)	0.007	0.570 (0.554-0.586)	0.008
GLR	0.567 (0.554-0.580)	0.007	0.576 (0.561-0.590)	0.007

Receiver Operating Characteristic (ROC) curves were used to individually assess predictive value of nutrition/inflammation-based indicators for mortality in sepsis patients. Area under the curve (AUC) and its 95% confidence interval (CI) were reported.

Markov Chain Monte Carlo (MCMC) multiple imputation methods are used to impute the missing values of nutrition/inflammation-based indices. Five imputations were established, tested, and reported.

ALI: advanced lung cancer inflammation index; GLR: glucose-to-lymphocyte ratio; GNRI: geriatric nutritional risk index; ICU: intensive care unit; LMR: lymphocyte-to-monocyte ratio; NLR: neutrophil-to-lymphocyte ratio; NPR: neutrophil-to-platelet ratio; PLR: platelet-to-lymphocyte ratio; PNI: prognostic nutritional index; SII: systemic immune-inflammation index; SIRI: systemic inflammation response index.

Supplementary Table 8: Predictive value of ten nutrition/inflammation-based indices for sepsis mortality in the MIMIC-IV 3.0 cohort after multiple imputation.

Nutrition/inflammation-based indices	Comparisons	Pooled results of original data and five multiple imputations [1]							
		In-hospital mortality [2]				In-ICU mortality [2]			
		Logistic regression models		Cox proportional hazards regression models		Logistic regression models		Cox proportional hazards regression models	
		OR (95% CI)	*P* value	HR (95% CI)	*P* value	OR (95% CI)	*P* value	HR (95% CI)	*P* value
NLR	Per 1 unit	1.016 (1.010-1.022)	<0.001	1.009 (1.007-1.012)	<0.001	1.014 (1.008-1.020)	<0.001	1.010 (1.007-1.014)	<0.001
PLR	Per 10 unit	1.000 (0.997-1.002)	0.77	1.000 (0.999-1.002)	0.88	0.999 (0.997-1.002)	0.54	1.000 (0.998-1.002)	0.87
NPR	Per 0.01 unit	1.007 (1.000-1.013)	0.035	1.006 (1.001-1.011)	0.012	1.005 (0.997-1.013)	0.19	1.004 (0.999-1.009)	0.095
LMR	Per 1 unit	0.987 (0.970-1.005)	0.15	0.996 (0.981-1.012)	0.62	0.992 (0.972-1.012)	0.40	0.995 (0.973-1.019)	0.67
SIRI	Per 1 unit	1.002 (0.997-1.007)	0.38	1.002 (0.998-1.007)	0.23	1.002 (0.996-1.008)	0.49	1.002 (0.996-1.008)	0.40
SII	Per 100 unit	1.001 (0.999-1.003)	0.30	1.001 (0.999-1.002)	0.29	1.002 (1.000-1.004)	0.11	1.001 (0.999-1.003)	0.44
PNI	Per 1 unit	1.001 (0.999-1.003)	0.45	1.001 (0.999-1.003)	0.19	1.001 (0.998-1.003)	0.71	0.999 (0.997-1.001)	0.51
GNRI	Per 1 unit	0.997 (0.994-0.999)	0.018	0.999 (0.997-1.002)	0.56	0.999 (0.996-1.002)	0.52	0.998 (0.996-1.001)	0.19
ALI	Per 1 unit	1.000 (0.999-1.000)	0.22	1.000 (1.000-1.000)	0.36	1.000 (0.999-1.000)	0.23	1.000 (1.000-1.000)	0.33
GLR	Per 100 unit	1.016 (1.003-1.029)	0.014	1.005 (1.002-1.008)	0.004	1.025 (1.011-1.038)	<0.001	1.008 (1.002-1.013)	0.005

[1] Markov Chain Monte Carlo (MCMC) multiple imputation methods are used to impute the missing values of nutrition/inflammation-based indices. Five imputations were established and tested. The pooled results of original data and five multiple imputations are presented.

[2] Ten nutrition/inflammation-based indices were simultaneously entered into multivariate analyses. Results are reported as odds ratios (ORs) or hazard ratios (HRs) with 95% confidence intervals (CIs).

ALI: advanced lung cancer inflammation index; GLR: glucose-to-lymphocyte ratio; GNRI: geriatric nutritional risk index; ICU: intensive care unit; LMR: lymphocyte-to-monocyte ratio; NLR: neutrophil-to-lymphocyte ratio; NPR: neutrophil-to-platelet ratio; PLR: platelet-to-lymphocyte ratio; PNI: prognostic nutritional index; SII: systemic immune-inflammation index; SIRI: systemic inflammation response index.
